# Shortwave‐Infrared‐Emitting Nanoprobes for CD8 Targeting and In Vivo Imaging of Cytotoxic T Cells in Breast Cancer

**DOI:** 10.1002/anbr.202300092

**Published:** 2023-12-05

**Authors:** Jay V. Shah, Jake N. Siebert, Xinyu Zhao, Shuqing He, Richard E. Riman, Mei Chee Tan, Mark C. Pierce, Edmund C. Lattime, Vidya Ganapathy, Prabhas V. Moghe

**Affiliations:** ^1^ Department of Biomedical Engineering Rutgers University 599 Taylor Rd Piscataway NJ 08854 USA; ^2^ Engineering Product Development Singapore University of Technology and Design 8 Somapah Rd Tampines Singapore 487372 Singapore; ^3^ Department of Materials Science and Engineering Rutgers University 607 Taylor Rd Piscataway NJ 08854 USA; ^4^ Rutgers Cancer Institute of New Jersey 195 Little Albany St New Brunswick NJ 08901 USA; ^5^ Department of Chemical and Biochemical Engineering Rutgers University 98 Brett Rd Piscataway NJ 08854 USA

**Keywords:** cytotoxic T cells, immune cell targeting, immunosurveillance, nanotechnologies, rare-earth probes, shortwave infrared imaging

## Abstract

Checkpoint immunotherapy has made great strides in the treatment of solid tumors, but many patients do not respond to immune checkpoint inhibitors. Identification of tumor‐infiltrating cytotoxic T cells (CTLs) has the potential to stratify patients and monitor immunotherapy responses. In this study, the design of cluster of differentiation (CD8^+^) T cell‐targeted nanoprobes that emit shortwave infrared (SWIR) light in the second tissue‐transparent window for noninvasive, real‐time imaging of CTLs in murine models of breast cancer is presented. SWIR‐emitting rare‐earth nanoparticles encapsulated in human serum albumin are conjugated with anti‐CD8α to target CTLs with high specificity. CTL targeting is validated in vitro through binding of nanoprobes to primary mouse CTLs. The potential for the use of SWIR fluorescence intensity to determine CTL presence is validated in two syngeneic mammary fat pad tumor models, EMT6 and 4T1, which differ in immune infiltration. SWIR imaging using CD8‐targeted nanoprobes successfully identifies the presence of CTLs in the more immunogenic EMT6 model, while imaging confirms the lack of substantial immune infiltration in the nonimmunogenic 4T1 model. In this work, the opportunity for SWIR imaging using CD8‐targeted nanoprobes to assess CTL infiltration in tumors for the stratification and monitoring of responders to checkpoint immunotherapy is highlighted.

## Graphical abstract

Identifying cytotoxic T cells (CTLs) in the tumor microenvironment can have implications for therapeutic response. In this study, shortwave‐infrared‐emitting nanoprobes are engineered to target CTLs with high specificity. CTL‐targeted nanoprobes enable noninvasive, real‐time imaging of tumor‐infiltrating CTLs and delineation of tumor immunogenicity in murine models of breast cancer.

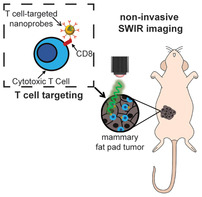

## 1 Introduction

Over the past decade, advances in oncological therapeutic strategies have led to the development of immune checkpoint inhibitors (ICIs). ICIs target and block immune checkpoints that would otherwise impede the ability of the immune system, particularly cytotoxic T cells (CTLs), to combat cancer.^[^ ^1^ ^]^ Currently, ICIs have been developed to target cytotoxic T lymphocyte antigen 4 (CTLA‐4), programmed cell death receptor 1 (PD‐1) or its ligand programmed cell death ligand 1 (PD‐L1), and lymphocyte activation gene 3.^[^ ^2^, ^3^ ^]^ Among these, ICIs that target the PD‐1/PD‐L1 axis have the most utility in treating many indications, either as standalone drugs or in combination with other forms of treatment, such as chemotherapy or other immunotherapies. For breast cancer, the combination of the anti‐PD‐1 inhibitor pembrolizumab and chemotherapy was approved in 2021 by Food and Drug Administration to treat metastatic and early‐stage triple‐negative breast cancer (TNBC).^[^ ^4^, ^5^ ^]^

Despite the improved therapeutic benefits of ICIs, most patients do not respond to the treatment, with response rates below 15%.^[^ ^6^, ^7^ ^]^ Monitoring immunotherapeutic responses is also confounded by pseudoprogression, a phenomenon characterized by an initial increase in tumor size due to increased inflammation followed by tumor regression.^[^ ^8^, ^9^ ^]^ Pseudoprogression can lead to the termination of effective immunotherapy.^[^ ^9^ ^]^ Alternatively, pseudoprogression can lead to ineffective therapy if misdiagnosed as tumor progression. Therefore, there is a critical need to identify biomarkers that can predict and monitor responses to ICIs. Although no definitive biomarker for ICI response prediction has been established, several have been shown to correlate to positive patient outcomes, including the presence of tumor‐infiltrating lymphocytes (TILs), PD‐L1 positivity, and tumor mutational burden.^[^ ^10^ ^]^ Tumors with high levels of TILs, especially cluster of differentiation (CD8^+^) CTLs, are generally associated with a good prognosis.^[^ ^11^ ^]^ Currently, TILs are assessed from biopsies and subsequent histological staining methods that do not allow for real‐time monitoring.^[^ ^12^ ^]^

Noninvasive imaging modalities using CD8‐targeted agents will allow for real‐time longitudinal monitoring of TILs in the tumor‐immune microenvironment (TIME). Recent studies combining positron emission tomography (PET) and radiolabeled antibodies^[^ ^13^, ^14^ ^]^ or antibody fragments^[^ ^15^, ^16^, ^17^ ^]^ have demonstrated the ability to identify TILs in small animal models of cancer. These probes have been utilized to track changes in immune infiltration in response to checkpoint immunotherapy.^[^ ^16^, ^17^, ^18^ ^]^ Clinical trials are underway to test this technology in humans.^[^ ^19^ ^]^ However, PET imaging is limited by the need for an anatomical reference, often requiring concurrent computed tomography imaging, increasing exposure to ionizing radiation and restricting the number of scans that can be performed.^[^ ^20^ ^]^

Optical imaging provides high spatial resolution and sensitivity without ionizing radiation.^[^ ^21^ ^]^ We have previously shown that human serum albumin (HSA)‐encapsulated rare‐earth (RE) nanoparticles (RENPs), termed RE albumin nanocomposites (ReANCs), when excited by a 980 nm near‐infrared (NIR) light source, emit shortwave infrared (SWIR) light, otherwise referred to as the second NIR optical window (NIR‐II; *λ* = 1000‐1700 nm).^[^ ^22^ ^]^ Compared to the light emitted by visible or NIR‐I (*λ* = 700–1000 nm) probes, SWIR light is less scattered by tissue, and the captured signal is not confounded by tissue autofluorescence, thus allowing for greater tissue penetration depth (up to 1 cm deep in a mouse) with high signal‐to‐noise ratios.^[^ ^23^, ^24^, ^25^ ^]^ Drug‐binding pockets and functional groups amenable to chemical conjugation on the albumin shell allow for functionalization, or targeting to disease‐specific cellular markers,^[^ ^26^, ^27^ ^]^ facilitating precision targeting and in vivo imaging of small cellular clusters.^[^ ^23^, ^24^ ^]^ Tumor‐targeted ReANCs have been shown to detect tumors earlier than imaging modalities such as MRI and can monitor therapy response in real time in small animal models of human cancer.^[^ ^22^, ^23^, ^24^, ^28^ ^]^

**Figure 1:**
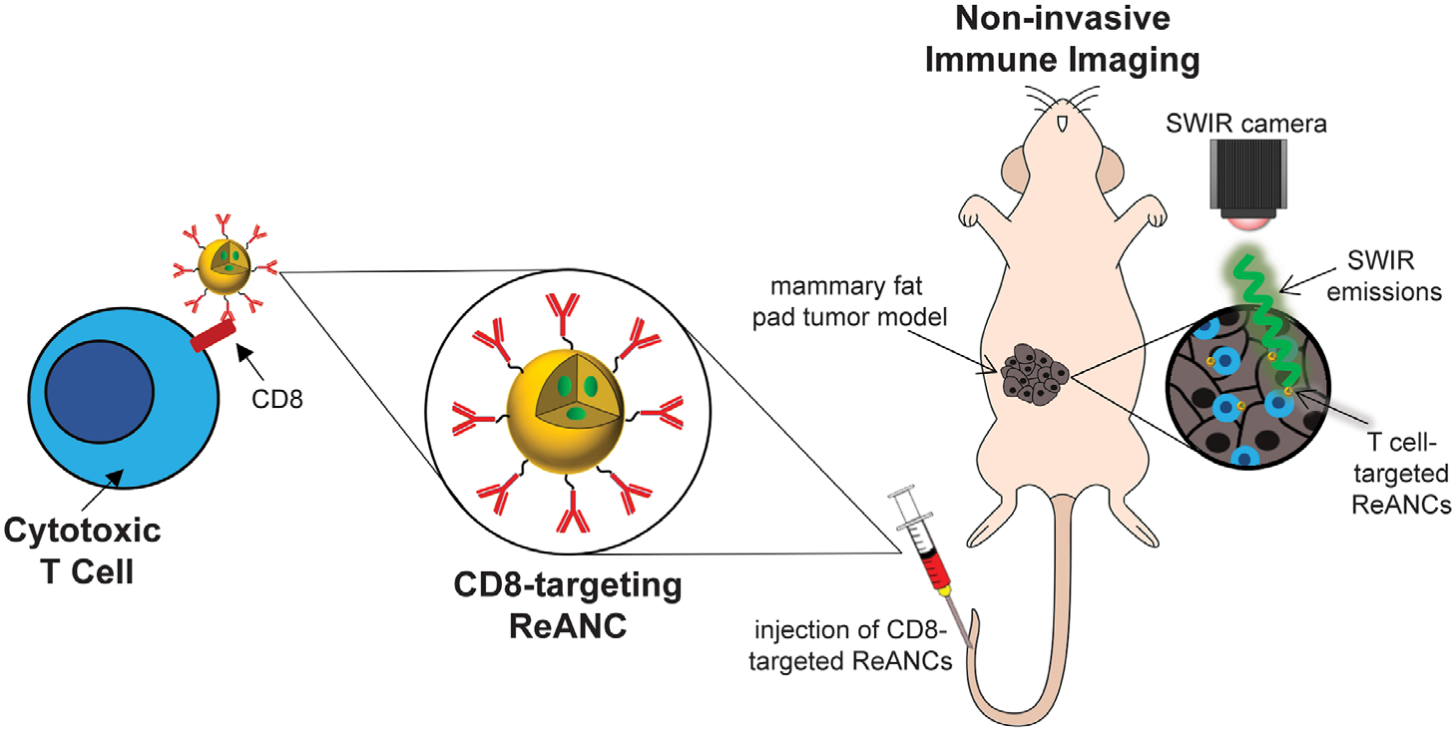
Non‐invasive shortwave infrared (SWIR) imaging of CD8^+^ T cells using rare‐earth (RE)‐encapsulated albumin nanocomposites (ReANCs). ReANCs decorated with anti‐CD8α antibodies facilitate T cell targeting via binding to the CD8 surface marker. Following an intravenous injection in a mouse bearing a mammary fat pad tumor, CD8‐targeted ReANCs accumulate in the immune‐infiltrated tumor. Whole‐body SWIR imaging using a 980 nm laser as an excitation source allows for imaging the ReANC‐labeled T cells within the tumor.

In this study, we explored the ability to use ReANCs to image tumor immunosurveillance in real time (**Figure** **1**). Using CD8‐targeting albumin nanocomposites (ANCs), we demonstrated specificity in vitro through binding to primary mouse CTLs with little to no secondary impact on the effector function of T cells. To differentiate the specificity of CD8‐targeted ReANCs in vivo, we utilized two murine TNBC mammary fat pad models, 4T1 and EMT6, that differ in immunogenicity, based on immune infiltration and responsiveness to immunotherapy.^[^ ^29^, ^30^, ^31^ ^]^ The 4T1 tumors are known to be nonimmunogenic, while EMT6 tumors are moderately immunogenic and respond modestly to checkpoint immunotherapy.^[^ ^29^, ^30^, ^31^, ^32^, ^33^, ^34^, ^35^ ^]^ The nonimmunogenic 4T1 model, with little CTL infiltration, had unremarkable differences in the SWIR signal intensities between CD8‐targeted or untargeted isotype control‐conjugated nanoprobes. In contrast, significantly greater SWIR signal was seen from CD8‐targeted ReANCs in the more immunogenic EMT6 tumors compared to control nanoprobes. We conclude that the findings from this study demonstrate the potential for assessing tumor‐immune burden, a plausible biomarker of immunotherapy response, through real‐time SWIR imaging using CD8‐targeted ReANCs.

## 2 Results and Discussion

### 2.1 In Vitro Characterization of CD8‐Targeted Nanocomposites

**Figure 2:**
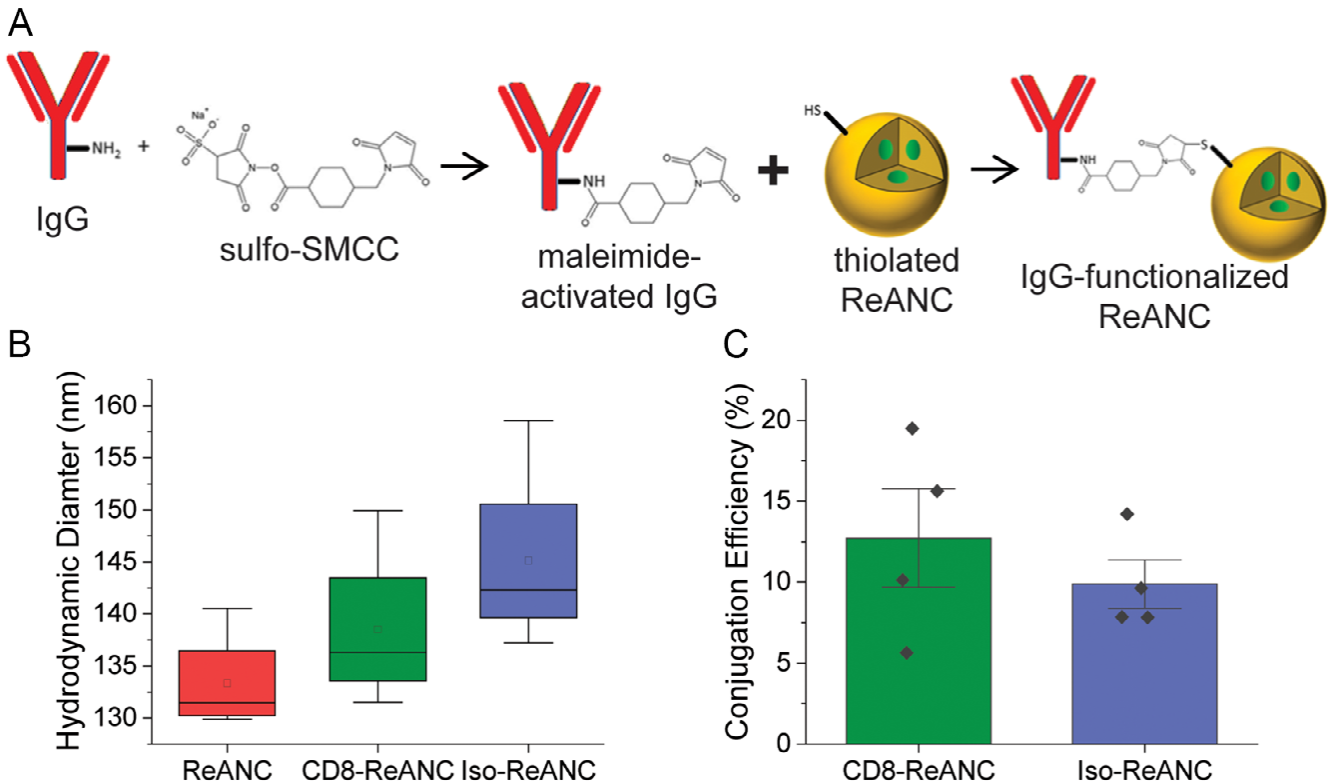
Engineering CD8‐targeting nanocomposites. A) The schematic illustrates the conjugation of immunoglobulin G (IgG) antibodies to albumin nanocomposite (ANCs). IgG activated with a maleimide group from sulfo‐SMCC reacts readily with thiol groups on the surfaces of thiolated ANCs and ReANCs, forming an uncleavable link between the IgG and ReANC. B) Hydrodynamic diameter of the nanocomposites (*n* = 4 per group), as assessed by dynamic light scattering. There was no significant difference between the size of CD8‐ and Iso‐ReANCs (*p* = 0.3232). C) The conjugation efficiency was evaluated using a direct enzyme‐linked immunosorbent assay (ELISA). There was no significant difference in the conjugation efficiencies of CD8‐ and Iso‐ReANCs (*p* = 0.4343). Groups were compared using a two‐tailed student's *t*‐test.

For in vitro target validation experiments, we used ANCs, as previous studies have shown that the presence of RENPs within the ANCs does not affect cellular interactions with the nanocomposites.^[^ ^26^ ^]^ For T cell targeting, the albumin shells of ANCs or ReANCs were functionalized with immunoglobulin G (IgG) antibodies as seen in **Figure** **2**A. The average hydrodynamic diameter of anti‐CD8α‐conjugated ReANCs (CD8‐ReANCs) was 138.5 ± 4.0 nm. ReANCs conjugated with the isotype control (Iso‐ReANCs) were 145.1 ± 4.6 nm in diameter. There were no significant differences in the hydrodynamic diameters or the polydispersity indices of CD8‐ and Iso‐ReANCs (Figure 2B, Table S1, Supporting Information). Unfunctionalized ANCs used in this study had a mean hydrodynamic diameter of ≈118 nm (117.9 ± 1.5 nm, *n* = 6) with a polydispersity index of 0.133±.03 before functionalization. ReANCs had a mean hydrodynamic diameter of 133.3 ± 2.4 nm and a polydispersity index of 0.141 ± .008 (*n* = 4) (Table S1, Supporting Information). The average nanocomposite yield from HSA was ≈96.6%, as assessed by a bicinchoninic acid (BCA) assay. As determined by enzyme‐linked immunosorbent assay (ELISA), the loading efficiencies were 12.7% ± 3.0% and 9.9% ± 1.5% for the anti‐CD8α and isotype control antibodies, respectively (Figure 2C). Based on loading efficiency, for nanocomposites used for in vivo imaging studies, this resulted in approximately 15–16 antibodies per nanocomposite (Table S2, Supporting Information) and an effective antibody dose of ≈16 μg per mouse (where 100 μL of ReANCs at a concentration of 50 μg HSA μL^−1^ were administered intravenously). This dose is similar to the amount of antibody used in published immunoPET imaging studies using CD8‐targeting PET probes in mice.^[^ ^14^, ^16^ ^]^

### 2.2 CD8‐Targeted ANCs Bind to Cytotoxic T Cells In Vitro with High Specificity

Previous studies have shown that while ReANCs can passively accumulate in tumors by the enhanced permeability and retention (EPR) effect,^[^ ^22^ ^]^ tumor‐targeting ReANCs have improved retention, enabling imaging of tiny clusters of cells.^[^ ^23^, ^24^ ^]^ Limiting factors for nanoprobe binding and accumulation in vivo include targeting ligand density and specificity.^[^ ^24^, ^36^ ^]^ ANCs labeled with fluorescein isothiocyanate (FITC) as an in vitro reporter were formulated with different amounts of antibody, ranging from 3.6 to 42.9 μg IgG mg^−1^ HSA, to determine an optimal ligand density.

**Figure 3:**
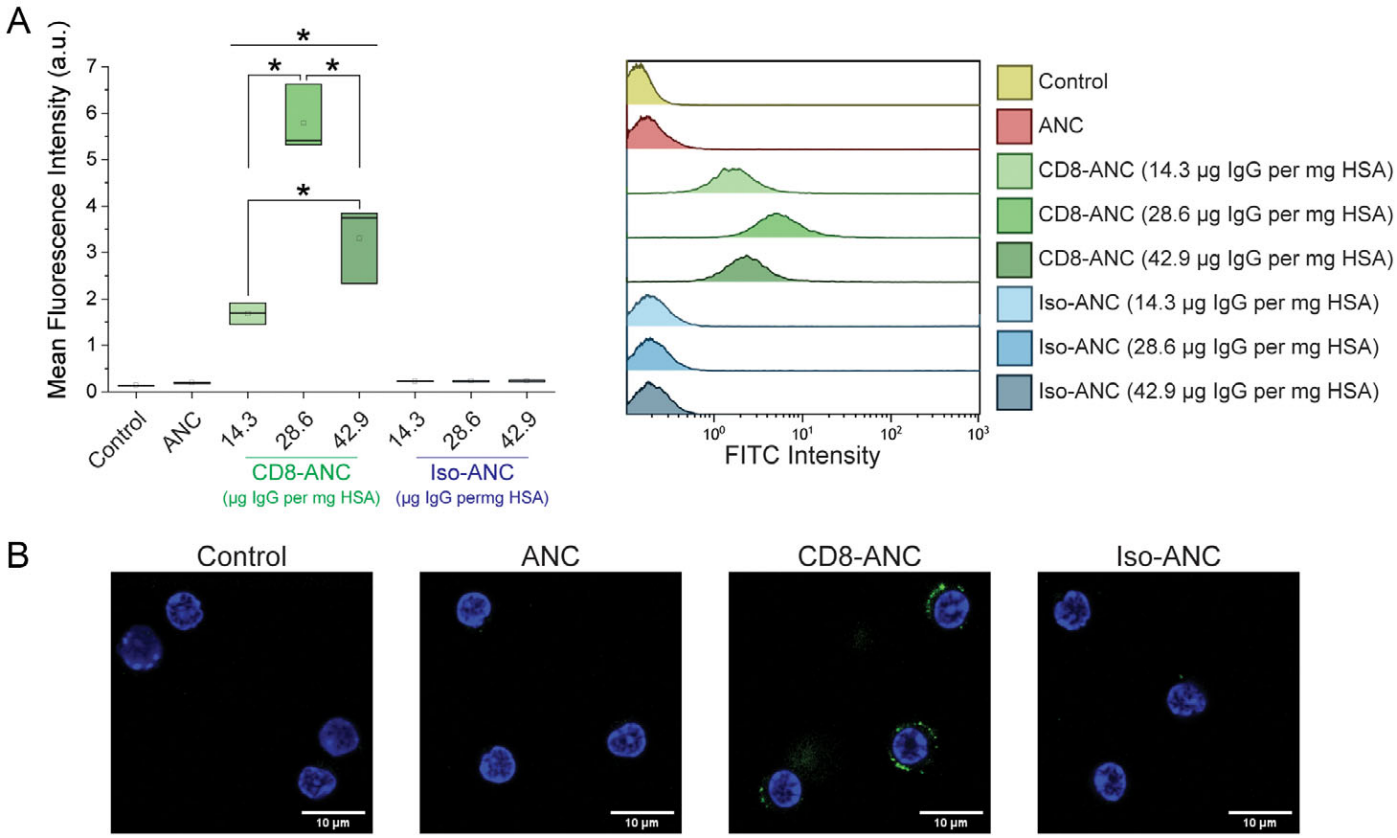
CD8‐targeting facilitates the uptake of ANCs by primary mouse cytotoxic T cells (CTLs). A) Conjugation of anti‐CD8α to ANCs increased binding to T cells as assessed by flow cytometry. Box plots (left) and histograms (right) demonstrate greater fluorescein isothiocyanate (FITC) fluorescence intensity from cells treated with CD8‐ANCs than nanocomposite‐free cell‐only controls, ANCs, and Iso‐ANCs. Among CD8‐ANCs, the highest nanocomposite uptake was achieved at the intermediate loading density of 28.6 μg IgG mg^−1^ HSA (*p* = 1.14 × 10^−7^ and *p* = 2.70 × 10^−5^ compared to loading densities of 14.3 and 42.9 μg IgG mg^−1^ HSA, respectively), while the group with the highest loading density of 42.9 μg IgG mg^−1^ had significantly greater MFI than the lowes*t* tested loading density of 14.3 μg IgG mg^−1^ (*p* = 0.0030). No increase in MFI was seen in the ANC or Iso‐ANC groups compared to the cell‐only control (*p* > 0.9999). The box plots represent the means from three independent experiments with three technical replicates per experiment. The histograms are representative of one of the three independent experiments. Analysis was performed using a one‐way analysis of variance (ANOVA) and a post hoc Tukey's test for multiple comparisons. Asterisks denote statistical significance. B) Representative fields of view from confocal imaging showed the presence of FITC‐labeled ANCs binding to the surface of T cells only following CD8‐ANC treatment. CD8‐ANCs and Iso‐ANCs used for imaging were functionalized using a loading density of 28.6 μg IgG mg^−1^ HSA. Scale bar = 10 μm.

Flow cytometry results demonstrated that CTLs treated with ANCs conjugated with the anti‐CD8α antibody (CD8‐ANCs) at any loading density had significantly greater mean fluorescence intensity (MFI) than untreated control cells and cells treated with unconjugated ANCs or ANCs conjugated with an isotype control antibody (Iso‐ANCs) (*p* < 0.01) (**Figure** **3**A and Figure S1, Supporting Information). This indicated a high binding affinity of CD8‐ANCs for their target CTLs. There was a nonlinear relationship between antibody loading and CD8‐ANC binding. This is consistent with similar studies that have reported nonlinear relationships between ligand density and target cell binding.^[^ ^24^, ^27^, ^36^ ^]^ Among the tested CD8‐ANCs, those conjugated with a loading density of 28.6 μg IgG mg^−1^ HSA had the most significant binding effect on CTLs, followed by a loading density of 42.9 μg IgG mg^−1^ HSA. We selected nanocomposites with the loading density of 28.6 μg IgG mg^−1^ HSA for all other experiments based on these results. For cells treated with control ANCs and Iso‐ANCs, there were insignificant differences in the MFI compared to the cell‐only negative control. Nanocomposite binding to CTLs was further validated by confocal imaging. As shown in Figure 3B, the FITC signal from CD8‐ANCs can be visualized around the periphery of the T cells. In contrast, for cells treated with control ANCs or Iso‐ANCs, there was little to no FITC signal visible around the cells.

The previous results demonstrate the targeting capability of CD8‐ANCs to murine CD8^+^ CTLs. To further validate the specificity of the CD8‐ANCs, uptake was tested with 4T1, EMT6, and Jurkat tumor cell lines that lack the CD8 marker. In the case of 4T1 and EMT6 mouse mammary carcinoma cells, there was no difference between the uptake of CD8‐ANCs and Iso‐ANCs for either cell type (Figure S2, Supporting Information). Furthermore, ex vivo immunohistochemistry staining of 4T1 and EMT6 tumor tissue with CD8‐ReANCs and Iso‐ReANCs showed an apparent increase in staining from the immunogenic EMT‐6 tumor sections when stained with CD8‐ReANCs compared to Iso‐ReANCs (Figure S3, Supporting Information). In contrast, the nonimmunogenic 4T1 tumor sections did not show appreciable staining with either one of the nanoprobes. Similarly, there was no difference in cellular uptake between the two nanocomposite types with Jurkat cells, a human CD8‐T cell line (Figure S4, Supporting Information). These results demonstrate enhanced specificity of the anti‐CD8α‐conjugated ANCs toward cells that primarily express CD8.

### 2.3 ANCs Do Not Impede T Cell Effector Functions

We tested the effect of CD8‐targeted nanocomposites on the viability and effector function of primary mouse T cells in vitro to ensure that the antibody binding does not lead to T cell inhibition.^[^ ^37^, ^38^, ^39^ ^]^ Typical in vivo depletion studies use antibody concentrations ranging from 100 μg to 1 mg of anti‐CD8 per mouse administered repeatedly over multiple days to eliminate CD8^+^ CTLs.^[^ ^15^, ^40^, ^41^, ^42^ ^]^ The effective dose of antibody injected per mouse in our in vivo studies was ≈16 μg as calculated by the loading efficiency determined by ELISA (Figure 2C), well below the amount needed for T cell depletion.

**Figure 4:**
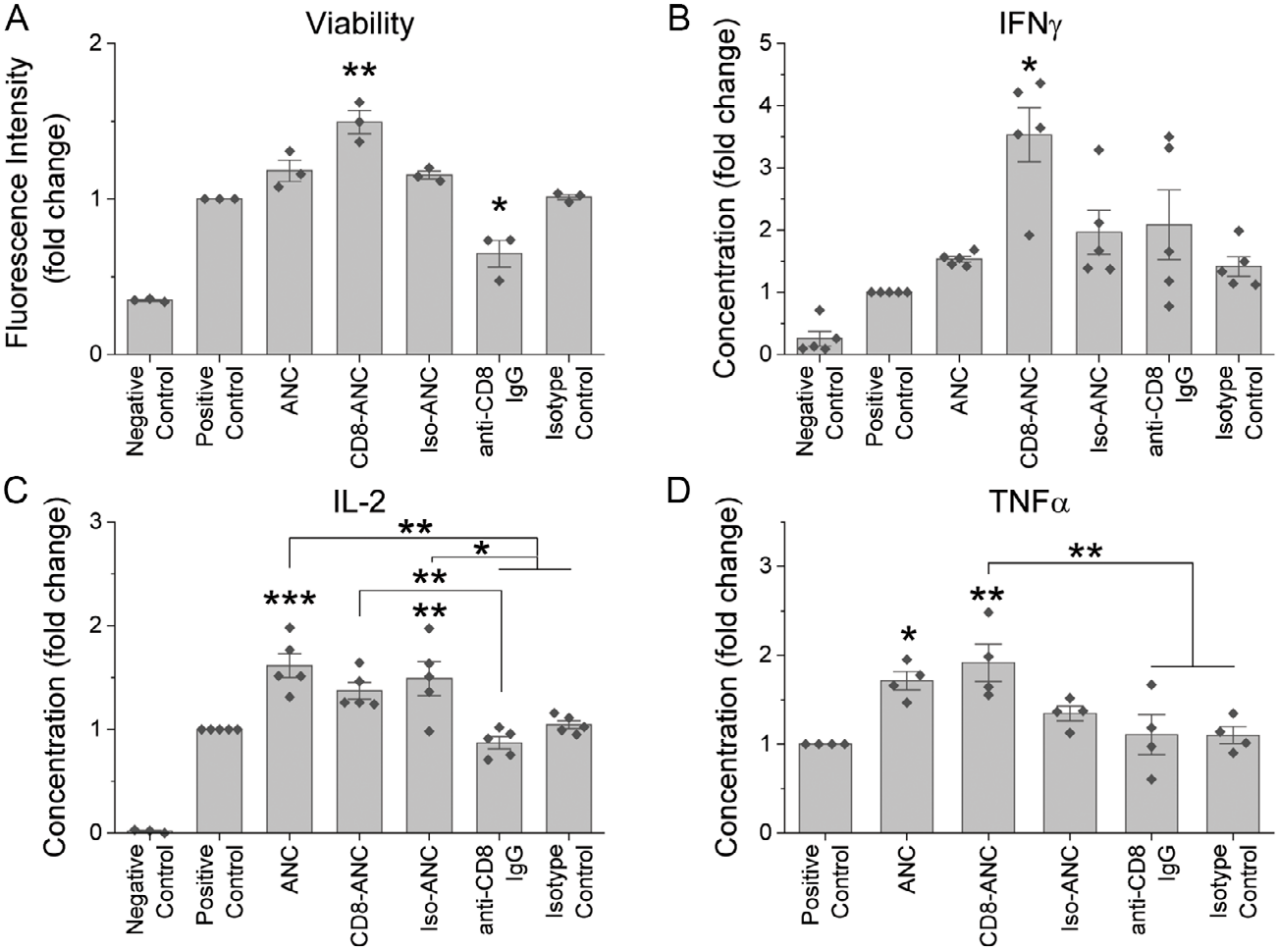
CD8‐targeted ANCs do not impede T cell functions in vitro. Primary mouse CTLs were activated with ConA and treated with ANCs or an equivalent concentration of soluble antibody for 24 h. The negative control consisted of CTLs that were not activated with ConA. A) Viability of CTLs was assessed by an alamarBlue cell viability assay (*n* = 3 per group). Cell viability was significantly greater after treatment with CD8‐ANCs than all other ConA‐treated groups (*p* < 0.01). CTLs treated with free anti‐CD8α IgG were significantly less viable than other ConA‐treated groups (*p* < 0.05). Cell culture supernatant collected after 24 h of treatment was analyzed by ELISA for B) IFNγ (*n* = 5 per group), C) IL‐2 (*n* = 5 per group; two experiments had negative controls that were below the level of detection), and D) TNFα (*n* = 4 per group; all negative controls were below the level of detection). Data is presented as the means from independent experiments with three technical replicates per group, normalized to the positive control. There was significantly more IFN‐γ in the supernatant of cells treated with CD8‐ANCs than other ConA‐treated groups. ANC and Iso‐ANC groups had a significantly greater concentration of IL‐2 than the positive control and free antibody groups, while CD8‐ANCs induced more IL‐2 secretion than soluble anti‐CD8α. ANC and CD8‐ANC groups had significantly greater TNF‐α levels than the positive controls, but only the CD8‐ANC group was greater than the free antibody groups. Statistical analysis was performed using a one‐way ANOVA with a post hoc Tukey's test for multiple comparisons. *p < 0.05, **p < 0.01, and ***p < 0.001.

Our studies on T cell viability showed that ANCs did not decrease the viability of concanavalin A (ConA)‐treated CTLs (**Figure** **4**A). Instead, we found an increase in cell viability with the CD8‐ANCs (*p* = 1.345 × 10^−4^) and a decrease with soluble anti‐CD8 IgG treatment (*p* = 0.0037), compared to the positive control. It has been shown that there are differences between soluble and immobilized antibodies when stimulating T cells in vitro.^[^ ^43^, ^44^ ^]^ Increased cell viability in this study with CD8‐ANCs compared to soluble anti‐CD8α could result from increased CD8 engagement and cross‐linking. Furthermore, there was no significant difference in viability among control T cells and T cells treated with CD8‐ANCs with varying ligand densities (Figure S5, Supporting Information).

Our studies of T cell cytokine profile posttreatment demonstrated that ANCs did not impede the effector functions of primary mouse CTLs. The concentrations of interferon (IFN)‐γ, interleukin (IL)‐2, and tumor necrosis factor (TNF)‐α in conditioned media of treated groups were at the same level or greater than the respective positive controls. T cells treated with CD8‐ANCs secreted significantly more IFN‐γ than the positive control (*p* = 6.06 × 10^−5^) (Figure 4B). Unfunctionalized ANC and Iso‐ANC treatment led to significantly higher IL‐2 secretion compared to the positive control (*p* = 7.48 × 10^−4^ and *p* = 0.0226, respectively), while CD8‐ANC treatment did not result in a statistically significant increase of IL‐2 (Figure 4C). Secreted TNF‐α levels (Figure 4D) for both the ANC and CD8‐ANC groups were significantly larger than the positive control (*p* = 0.0226 and *p* = 0.0027, respectively). The results from these experiments show that ReANCs can be used as nanoprobes to image CTLs with little to no impact on their effector function.

We observed that cytokine levels were significantly greater with CD8‐ANC treatment than with soluble anti‐CD8α treatment. This is consistent with the viability data and could be due to increased CD8 engagement and cross‐linking.

### 2.4 4T1 and EMT6 Mammary Fat Pad Tumors Differ in Immunogenicity but Not Growth Patterns

To demonstrate the differential specificity of CD8‐targeted nanocomposites in vivo, we used two mammary fat pad tumor models that differ in immunogenicity. The 4T1 tumors are immunologically cold and are known to have low lymphocyte infiltration and poor therapeutic responses to ICIs.^[^ ^32^, ^45^, ^46^ ^]^ Conversely, EMT6 tumors are more immunogenic^[^ ^30^, ^34^, ^47^ ^]^ and have been shown to respond to checkpoint immunotherapy.^[^ ^29^, ^31^, ^33^, ^35^ ^]^ Responsiveness of EMT6 tumors to immune checkpoint therapy has been attributed to the presence of CTLs since the depletion of CD8^+^ T cells diminishes the response.^[^ ^31^, ^33^, ^48^ ^]^ Immunogenicity based on CTL infiltration was validated by immunohistochemistry (Figure S6, Supporting Information). CD8^+^ staining was present in both tumor models; however, in EMT6 tumors, there were areas of high CD8 density, particularly at the invasive margins of the tumors. In 4T1 tumors, CD8 staining was more sporadic in the tumor core, with little to no staining at the invasive margins. Although they differed in immunogenicity, the tumor models had similar growth patterns with no significant differences (Figure S7, Supporting Information).

### 2.5 CD8‐Targeted ANCs Demonstrate Specificity In Vivo and Allow for T Cell SWIR Imaging

CTLs have been shown to have the potential to serve as biomarkers for immunotherapy response. The ability to identify the existence or lack of a CTL population can have implications for stratifying patients into responder and nonresponder groups for checkpoint immunotherapy.^[^ ^49^ ^]^ CTLs are assessed in the clinic through immunohistochemistry staining of T cell markers in biopsy specimens,^[^ ^12^ ^]^ and thus cannot provide real‐time information. This study was focused on using CD8‐targeted SWIR‐emitting nanoprobes to assess CD8 immune burden in real time in two syngeneic mammary fat pad models of breast cancer: 4T1 and EMT6.

**Figure 5:**
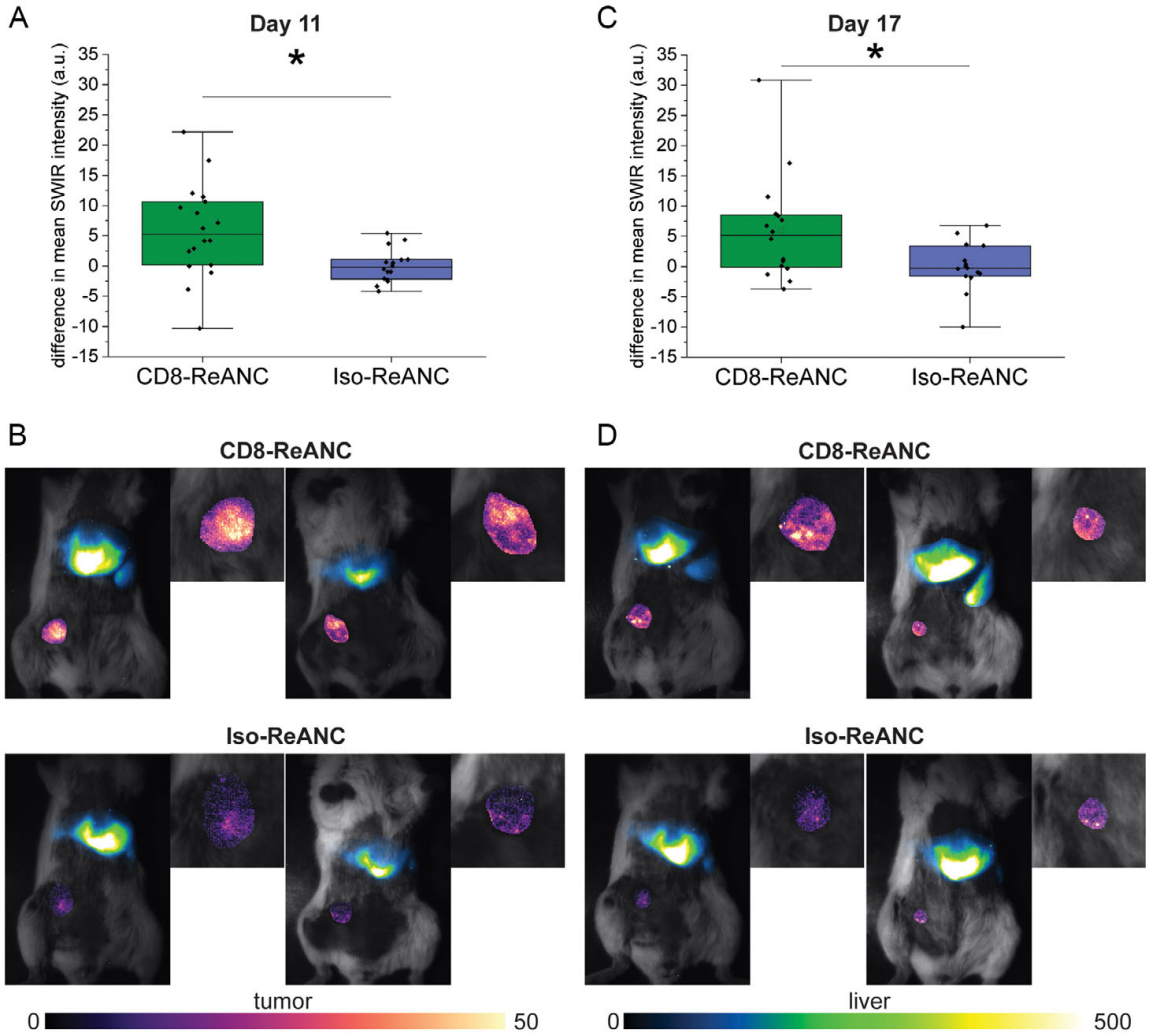
CD8‐targeted ReANCs facilitate noninvasive, in vivo imaging of tumor‐associated T cells in EMT6 tumor‐bearing mice. Mice were inoculated with 4 × 10^5^ EMT6 breast cancer cells in the fourth right teat and SWIR imaged after intravenous injections of CD8‐ or Iso‐ReANCs 11 and 17 days after tumor inoculation. A) Quantification of the SWIR signal in the tumors of mice showed more accumulation of CD8‐ReANCs compared to Iso‐ReANCs (*p* = 0.0120) over three independent experiments. B) Representative images of two mice per group on day 11 demonstrate the differences in SWIR signal from tumors that received CD8‐ReANCs (top) and Iso‐ReANCs (bottom). C) Significant differences between CD8‐ReANC and Iso‐ReANC SWIR signals in the tumors were observed on day 17 (*p* = 0.0119) over three independent experiments. D) Representative images of two mice per group reveal differences in the SWIR signal intensity in the EMT6 tumors on day 17. Data points in the box plots represent the difference between the mean pixel intensity from each tumor and the mean SWIR intensity from the Iso‐ReANC group for each experiment. Statistical analysis was performed by first performing a student's *t* test (with Welch's correction if variances were unequal) for each experiment, followed by a weighted Z‐score to compare the results across the three independent experiments. Data from individual experiments is provided in Figure S7, Supporting Information. *p < 0.05.

We observed significant differences in the SWIR signal of CD8‐ReANCs and Iso‐ReANCs in EMT6 tumors on day 11 postinoculation (**Figure** **5**A). The mean SWIR intensity from CD8‐ReANCs was greater than Iso‐ReANCs, with a *p*‐value of 0.0120 across three experiments (*p* = 0.0490, *p* = 0.4517, and *p* = 0.0822 for individual experiments [Figure S8, Supporting Information]). Representative images show that the tumor signal was relatively brighter for mice that received CD8‐ReANCs (Figure 5B). Similarly, we saw significant differences between the two nanocomposite formulations in EMT6 tumors on day 17 postinoculation (Figure 5C,D). The *p*‐value across all three experiments was 0.0119 (*p* = 0.0172, *p* = 0.0822, and *p* = 0.6166 for individual experiments), suggesting that the ability to target and image CTLs in the EMT6 model is not dependent on tumor size. These observations highlight the power of SWIR imaging using CD8‐targeted ReANCs for real‐time evaluation of CD8 burden; such insights could guide decision‐making in regimen alterations early during immunotherapy.

**Figure 6:**
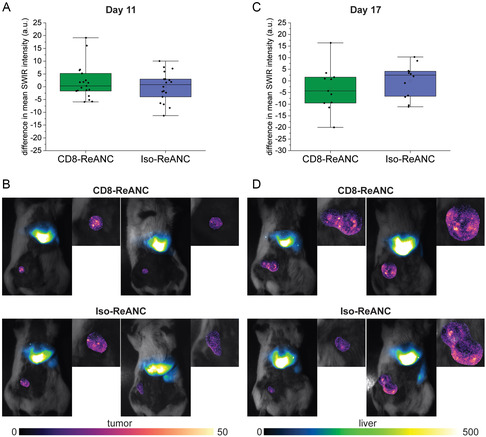
CD8‐targeted ReANCs do not detect T cell burden in the immunologically cold 4T1 tumor model. Mice were inoculated with 4 × 10^5^ 4T1 breast cancer cells in the fourth right teat and SWIR imaged after intravenous injections of CD8‐ or Iso‐ReANCs 11 and 17 days after tumor inoculation. A) No differences were observed in the quantifiable SWIR signals of mice that received CD8‐ReANCs compared to Iso‐ReANCs across three independent experiments (*p* = 0.4954). B) Qualitative analysis of SWIR images of two mice per group also shows no appreciable differences between CD8‐ReANCs (top) and Iso‐ReANCs (bottom). C) SWIR imaging and quantification on day 17 also showed no difference between either administered ReANC type (*p* = 0.5284). D) Representative SWIR images on day 17 show no qualitative differences between the tumors of mice that received CD8‐ReANCs (top) and Iso‐ReANCs (bottom). Data points in the box plots represent the difference between the mean pixel intensity from each tumor and the mean SWIR intensity from the Iso‐ReANC group for each experiment. Statistical analysis was performed by first performing a student's *t‐*test (with Welch's correction if variances were unequal) for each experiment, followed by a weighted Z‐score to compare the results across the three independent experiments. Data from individual experiments is provided in Figure S8, Supporting Information.

In contrast, in the immunologically cold 4T1 tumors, no differences were observed in SWIR signal between CD8‐ReANCs and Iso‐ReANCs across the three experiments (**Figure** **6**) (*p* = 0.4954 for day 11 [*p* = 0.9633, *p* = 0.3562, and *p* = 0.8568 for individual experiments (Figure S9, Supporting Information)] and *p* = 0.5284 for day 17 [*p* = 0.9938, *p* = 0.4168, and *p* = 0.8407 for separate experiments]). The lack of a difference between SWIR intensities in 4T1 tumor‐bearing mice treated with CD8‐ReANCs and Iso‐ReANCs indicates nonspecific binding or passive uptake of the nanocomposites.

The ability to detect the immune population around differentially immunogenic tumors demonstrates the potential of SWIR‐emitting ReANCs to stratify tumors based on their immunological state. However, the high nonspecific uptake in the 4T1 tumors suggests that further molecular‐level discrimination may be necessary to fully decouple the specific versus nonspecific uptake of the nanoprobes between the 4T1 and EMT6 models. SWIR‐emitting nanocomposites have multispectral capabilities based on the RE dopant.^[^ ^22^, ^27^ ^]^ Future studies can address the issue of delineating signal from nonspecific binding through multispectral imaging using a mix of differently doped CD8‐targeted and isotype control‐conjugated ReANCs to unravel the actual signal from that of nonspecific uptake through spectral unmixing.^[^ ^50^ ^]^

CD8‐targeted ReANCs can be used for monitoring of genetically engineered immune cells in an adoptive cellular therapy setting and track their trafficking to immunogenic tumors. In future studies, ex vivo manipulated T cells can be labeled with CD8‐ReANCs ex vivo to monitor the in vivo fate of adoptive T cell transfer using optical imaging.

### 2.6 CD8‐Targeted ANCs Can Be Administered Safely and Do Not Elicit Noticeable Damage in Organs of Clearance

Previous studies in athymic nude mice have demonstrated that ReANCs clear through both the liver and spleen.^[^ ^23^ ^]^ In the syngeneic mouse models, this study observed similar clearance patterns through liver and spleen. Iso‐ReANCs appeared to accumulate more frequently than CD8‐ReANCs in the liver (*p* = 0.0231 for 4T1 and *p* = 0.0260 for EMT6) (Figure S10, Supporting Information). This data is aligned with our previous observation that targeted ReANCs (CD8‐ReANCs) have increased retention in the target tissue of interest (i.e., CD8^+^ T cells). Furthermore, there was no significant difference in tumor growth observed between mice that received CD8‐ReANCs or Iso‐ReANCs (Figure S11, Supporting Information).

Hematoxylin and eosin (H&E) staining of livers of mice treated with CD8‐ReANCs or Iso‐ReANCs showed no apparent nanocomposite‐induced damage compared to untreated controls (Figure S12, Supporting Information). All livers displayed standard hepatic architecture, with some evidence of tumor invasion in the livers of 4T1‐bearing mice. The spleens of EMT6 mice showed typical splenic architecture with well‐defined white pulp regardless of nanocomposite injection, while 4T1 mice showed reduced white pulp and the presence of large macrophages (Figure S13, Supporting Information). However, this could be because splenomegaly and increased splenic granulocytes are common occurrences in the 4T1 model.^[^ ^51^, ^52^ ^]^

This study in EMT6 and 4T1 tumor‐bearing mice highlights the potential for ReANCs to be engineered to target other T cell subsets, such as helper T cells or pro‐tumorigenic regulatory T cells. In addition to erbium‐based RENPs, our lab has demonstrated the ability to utilize holmium‐ and thulium‐doped RENPs for small animal in vivo imaging.^[^ ^27^ ^]^ These RE cores possess distinct emission profiles, paving the way for multispectral imaging of multiple target epitopes.^[^ ^27^ ^]^ It has been suggested that the spatial distribution and ratios of CD8^+^ and CD4^+^ T cells within the tumor can provide more prognostic information than tumor‐infiltrating CTLs alone.^[^ ^10^ ^]^ Another confounding factor in immunotherapy responsiveness is the presence of CD8^+^ T cells with an exhausted phenotype.^[^ ^53^, ^54^ ^]^ The ability to delineate the immune cells in the tumor microenvironment based on their ability to mount effector function and using the ratio to exhausted T cells as a metric for real‐time therapy response monitoring could be very attractive and beneficial. Future work from this group will address the ratiometric analysis of the different immune subsets and their correlation to therapy response.

## 3 Conclusion

In summary, we have engineered SWIR‐emitting nanocomposites to target and image CD8^+^ cytotoxic T cells in vivo. We demonstrated high specificity and affinity of the nanocomposites toward primary mouse CTLs in vitro without impeding the ability of T cells to proliferate or secrete antitumor cytokines. CD8‐targeted ReANCs were tested for in vivo specificity using two models of murine TNBC that are known to differ in immunogenicity and respond to checkpoint immunotherapy. We showed that the SWIR signal from the more immunogenic EMT6 tumors of mice administered CD8‐ReANCs was significantly greater than that of mice receiving ReANCs conjugated with an isotype control antibody. Meanwhile, no differences were observed between ReANC formulation and nonimmunogenic 4T1 tumors. The work presented here demonstrates proof of concept for the use of ReANCs and SWIR imaging to noninvasively image tumor‐associated immune cells, with the potential to utilize this technology to monitor dynamic changes in the immunogenic state of murine mammary fat pad tumors after administration of ICIs.

## 4 Experimental Section

4.1

4.1.1

##### Synthesis of REANCs

As previously described, RE‐metal‐doped nanoparticles were synthesized by burst nucleation reaction.^[^ ^22^, ^55^ ^]^ The RENPs contained NaYF_4_ cores doped with ytterbium (Yb) and erbium (Er) and undoped NaYF_4_ shells. ANCs and ReANCs, containing RENPs encapsulated by HSA, were synthesized as previously reported using solvent‐induced controlled coacervation.^[^ ^22^, ^23^, ^24^ ^]^ Briefly, HSA (20 mg mL^−1^) (Millipore Sigma, Burlington, MA) was dissolved in 10 mm NaCl and adjusted to a pH of 8.50 ± 0.05 with 0.1 m NaOH. RENPs (0.4 mg mL^−1^) were dissolved in 100% ethanol and dispersed in an ultrasonic bath for 1 h. Pure ethanol was used to make ANCs devoid of RENPs. To incorporate a fluorescent dye for in vitro experiments, FITC (12.6 μg mL^−1^) was dissolved in the ethanol solution. Using a syringe pump with an infusion rate of 1.5 mL min^−1^, RE solution or ethanol (2 mL) was added to HSA solution (500 μL) in a 20 mL scintillation vial under continuous stirring at 1000 RPM on a 2mag MIX 15 eco magnetic stirrer (Millipore Sigma). Following infusion, 8% glutaraldehyde was added to the vial at a ratio of 0.234 μL mg^−1^ HSA, and the solution was allowed to stir for 16–18 h. ANCs or ReANCs were then purified by two rounds of centrifugation (Avanti J‐E centrifuge, Beckman Coulter, Brea, CA) at 48 400 × g for 10 min at 4 °C. The nanocomposites were resuspended in phosphate‐buffered saline (PBS) + 1 mM ethylenediaminetetraacetic acid (EDTA) at 5 mg HSA mL^−1^ concentration for further functionalization.

##### Thiolation of ANCs

The albumin shells of ANCs or ReANCs were first thiolated using Traut's reagent (2‐iminothiolane), which adds a sulfhydryl group to a free amine.^[^ ^56^ ^]^ Traut's reagent (2 mg mL^−1^) (Thermo Fisher Scientific, Waltham, MA) was dissolved in water and added to ANCs or ReANCs in PBS + 1 mm EDTA at a ratio of 2 mg Traut's reagent per 17.5 mg HSA. The solution was placed on a Benchmixer™ XL multi‐tube votexer shaker (Millipore Sigma) for 1 h with shaking at 600 RPM. The nanocomposites were then purified by centrifugation at 48 400 g for 10 min at 4 °C and resuspended in PBS + 1 mM EDTA at a concentration of 5 mg HSA mL^−1^ for conjugation with antibodies.

##### Functionalization of ANCs with Antibodies

ANCs or ReANCs were functionalized with antibodies using maleimide chemistry.^[^ ^56^ ^]^ To target murine CTLs, anti‐mouse CD8α rat IgG2a (Clone 53‐6.7, Bio X Cell, Lebanon, NH) was conjugated to ANCs and ReANCs. As an isotype control, anti‐trinitrophenol (Clone 2A3, Bio X Cell) was used in experiments as a biologically irrelevant target. Both antibodies will be referred to as IgG to describe functionalization. Briefly, ANCs or ReANCs (5 mg HSA mL^−1^) were prepared in PBS + 1 mm EDTA (3.5 mL). Sulfo‐succinimidyl 4‐(N‐maleimidomethyl)cyclohexane‐1‐carboxylate (sulfo‐SMCC; 5 mg mL^−1^) (Thermo Fisher Scientific) was dissolved in water. Sulfo‐succinimidyl 4‐(N‐maleimidomethyl)cyclohexane‐1‐carboxylate (SMCC) was added to a solution of IgG in PBS + 1 mm EDTA at a 20‐fold molar excess and reacted for 30 min at room temperature to activate the IgG with a maleimide group. Excess sulfo‐SMCC was separated from the maleimide‐activated IgG by filtration using an Amicon Ultra 10 000 kDa molecular weight cutoff centrifugal filter unit (Millipore Sigma) spun at 14 000 g for 15 min. For in vivo experiments, the maleimide‐activated IgG was adjusted to a concentration of 1 mg mL^−1^ in PBS + 1 mm EDTA and added to ANCs or ReANCs at a ratio of 28.6 μg IgG mg^−1^ HSA. For in vitro experiments testing the effects of loading density on T cell binding, the volume of maleimide‐activated IgG was adjusted to maintain the volume ratio of 500 μL IgG to 3.5 mL ANC. Maleimide‐activated IgG and nanocomposites were reacted for 30 min at room temperature on a Benchmixer XL multi‐tube votexer shaker set at 600 RPM. The nanocomposites were centrifuged at 48 400 g for 10 min at 4 °C and resuspended in PBS at a final concentration of 50 mg HSA mL^−1^.

##### Characterization of Nanocomposites

Nanocomposite yield before functionalization was determined from the supernatants obtained during purification. According to manufacturer's instructions, the total protein content from the supernatant was estimated using a BCA assay (Thermo Fisher Scientific). The size and polydispersity of nanocomposites were determined with dynamic light scattering in a DynaPro plate reader (Wyatt Technology, Santa Barbara, CA). The hydrodynamic diameters and polydispersity indices were measured before and after IgG conjugation.

##### Assessment of Antibody Conjugation to Nanocomposites

The amount of antibody conjugated to ANCs or ReANCs was quantified by a direct ELISA. A standard curve of free IgG was prepared to start from 20 ng mL^−1^ and serially diluted. ReANCs were diluted to a 1.25 μg HAS mL^−1^ concentration, with a theoretical maximum of 35.7 μg mL^−1^ of conjugated IgG. ReANCs that were not functionalized were used as negative controls. Diluted ReANCs or IgG standard (100 μL) were plated in triplicate in Nunc Maxisorp 96‐well plates (Thermo Fisher Scientific) and incubated overnight at 4 °C. The plate was then washed three times with PBS + 0.05% Tween. At room temperature, the wells were blocked with 1% bovine serum albumin (BSA) in PBS (200 μL) for 1 h. The plate was washed three more times with PBS + 0.05% Tween. Next, goat anti‐rat IgG heavy and light chains (H&L) conjugated with horseradish peroxidase (HRP) (ab205720, Abcam, Waltham, MA) was prepared in 1% BSA (20 ng mL^−1^), added to each well (100 μL), and incubated for 2 h at room temperature to detect the presence of rat IgG2a. Following three more washes with PBS + 0.05% Tween, a solution of stabilized hydrogen peroxide and stabilized tetramethylbenzidine (R&D Systems, Minneapolis, MN) was added to each well (100 μL), producing a blue color in the presence of HRP. After 5 min, the reaction was stopped with 2 n sulfuric acid (50 μL). The absorbance (*λ* = 450 nm) from each well was measured using a microplate reader (Tecan, Switzerland) with a reference (*λ* = 570 nm). The optical density (OD) from the standards was plotted on a log–log graph, and a standard curve was calculated. The OD from the nanocomposites was compared against the standard curve to estimate the number of antibodies conjugated to the nanocomposites.

##### Cell Culture

The 4T1 and EMT6 mouse mammary cancer cells were purchased from American Type Culture Collection (Old Town Manassas, VA). The 4T1 cells were cultured in Dulbecco's Modified Eagle Medium (DMEM) (Gibco, Waltham, MA) supplemented with 10% fetal bovine serum (FBS) (Thermo Fisher Scientific) and 1% penicillin/streptomycin (P/S) (Thermo Fisher Scientific). EMT6 cells were cultured in Waymouth's MB 752/1 media (Gibco) supplemented with 10% FBS and 1% P/S. Jurkat cells were a kind gift from Dr. Jeffrey Zahn and cultured in Roswell Park Memorial Institute (RPMI) 1640 media (Gibco) supplemented with 2 mm L‐glutamine, 10 mm 4‐(2‐hydroxyethyl)‐1‐piperazineethanesulfonic acid (HEPES), 1 mm sodium pyruvate, 4500 mg L^−1^ glucose, 1500 mg L^−1^ sodium bicarbonate, 10% FBS, and 1% P/S. Cells were incubated at 37 °C in a humidified atmosphere of 5% CO_2_.

##### Animals

All animal studies performed in this report were approved under Protocol ID999900469 by the Institutional Animal Care and Use Committee of Rutgers University and performed in accordance with institutional guidelines on animal handling. BALB/c mice were purchased from Charles River Laboratories (Wilmington, MA) and provided with food and water ad libitum. The animals were maintained in a controlled environment of 23 °C, 35% humidity, and a 12 h light–dark cycle.

##### Isolation of Primary Mouse Cytotoxic T Cells

Spleens were resected from 6–8‐week‐old female BALB/c mice, manually crushed, and filtered through a 40 μm nylon cell strainer to obtain a single cell suspension of splenocytes. The splenocytes were resuspended in PBS + 2% FBS + 1 mm EDTA at a concentration of 1 × 10^8^ cells mL^−1^. According to the manufacturer's instructions, primary cytotoxic T cells were isolated from the splenocytes using an EasySep mouse CD8^+^ T cell negative isolation kit (Stemcell Technologies, Vancouver, BC, Canada).

##### Assessment of Nanocomposite Binding by Flow Cytometry

The binding of CD8‐targeted ANCs to CTLs was assessed via flow cytometry. Primary mouse CTLs isolated from BALB/c mice (1 × 10^6^ cells mL^−1^) were resuspended in RPMI 1640 media (Gibco) supplemented with 10% FBS and 1% P/S. The cell suspension (100 μL) was plated into U‐bottom 96‐well plates. Cells were then treated with 10 μL of PBS as a cell‐only control or FITC‐labeled ANCs (0.5 mg HSA mL^−1^) with varying ligand density. The T cells were then incubated for 1 h at room temperature with gentle shaking. The cells were then centrifuged and washed three times with cold PBS. After the final wash, T cells were resuspended in cold PBS (300 μL) and incubated with DAPI for live cell flow cytometry analysis on a Gallios flow cytometer (Beckman Coulter). The data were gated based on the cell‐only control, and nanocomposite binding was assessed by FITC fluorescence. Experiments were run three times with technical triplicates. The data were analyzed using a one‐way analysis of variance (ANOVA) with a Tukey's *post hoc* test for multiple comparisons. For uptake experiments with tumor cell lines, 4T1 cells (5 × 10^4^ cells) in complete DMEM or EMT6 cells (5 × 10^4^ cells) in complete Waymouth's media were plated in 96‐well plates and attached overnight. The cells were washed and treated with PBS as a cell‐only control or FITC‐labeled ANCs (0.5 mg HSA mL^−1^) for 24 h. The wells were then washed, the cells were trypsinized, and the cells were fixed with 1% paraformaldehyde (PFA) in PBS for analysis by flow cytometry.

##### Assessment of Nanocomposite Binding by Confocal Microscopy

To assess nanocomposite binding by confocal microscopy, primary mouse CTLs isolated from BALB/c mice (1 × 10^6^ cells mL^−1^) were resuspended in RPMI 1640 media supplemented with 10% FBS and 1% P/S. The T cell suspension (100 μL) was treated with PBS‐ or FITC‐labeled ANCs (0.5 mg HSA mL^−1^). The cells were incubated for 1 h at room temperature with gentle shaking. Cells were washed two times with cold PBS and stained with Hoechst 33 342 (10 μg mL^−1^) (Thermo Fisher Scientific). Cells were rewashed with PBS and fixed with 1% PFA for 10 min. The fixed cells were rewashed with PBS, resuspended in PBS (100 μL), and transferred to a Lab‐Tek 8‐well chambered cover glass (Thermo Fisher Scientific). The chamber slide was imaged with a Zeiss LSM 780 confocal laser scanning microscope (Carl Zeiss AG, Germany). A 405 nm laser line was used to excite the Hoechst stain to visualize nuclei, and a 488 nm laser line was used to excite FITC to visualize ANCs.

##### Assessment of T Cell Effector Function Following Nanocomposite Treatment by ELISA

To ensure the effector function of CTLs after ANC treatment, conditioned media from activated T cells was probed for release of cytokines by ELISA. Primary mouse CTLs isolated from BALB/c mice (1 × 10^6^ cells mL^−1^) were resuspended in RPMI 1640 media supplemented with 10% FBS and 1% P/S. T cells were activated with concanavalin A (ConA; 2.5 μg mL^−1^) (Thermo Fisher Scientific). T cells without ConA treatment served as a negative control. T cells (1 × 10^5^ cells) were plated in U‐bottom 96‐well plates and treated with 10 μL of PBS for the negative and positive controls, ANCs, CD8‐ANCs, Iso‐ANCs, anti‐CD8α IgG (Clone 53‐6.7), or isotype control IgG (Clone 2A3). ANC treatment was performed at a nanocomposite concentration of 0.5 mg HSA mL^−1^. Free antibody treatment was performed at an equivalent concentration to match the amount of antibody used during the functionalization of CD8‐ANCs and Iso‐ANCs. The plate was then incubated at 37 °C for 24 h. The cells were centrifuged, and the supernatant was collected from the cells and stored at −80 °C until used for ELISA. The conditioned media was probed for the secretion of IFN‐γ, IL‐2, and TNF‐α from activated T cells using mouse IFN‐γ, IL‐2, and TNF‐α DuoSet ELISA kits (R&D Systems), respectively, according to manufacturer's instructions.

##### Viability Assay Following Nanocomposite Treatment

T cell viability after ANC treatment was performed with an AlamarBlue assay (Thermo Fisher Scientific) according to the manufacturer's instructions. Primary mouse CTLs treated with ANCs were washed with PBS and then treated with AlamarBlue HS cell viability reagent diluted in RPMI 1640 media supplemented with 10% FBS and 1% P/S for 1 h at 37 °C. The fluorescence of the wells following incubation was read (*λ*_Ex_ = 560 nm, *λ*_Em_ = 590 nm) and compared to control T cells that did not receive ANC treatment.

##### Tumor Models

Six‐week‐old, female BALB/c mice were purchased from Charles River Laboratories and allowed to acclimate to laboratory conditions. One day before mammary fat pad tumor inoculation, the fur around the fourth right teat of the mouse was removed with Nair hair removal cream and thoroughly cleaned to prevent skin irritation. To establish the tumor models, mice were anesthetized with 2%–3% isoflurane (Henry Schein, Melville, NY) in oxygen. The 4T1 or EMT6 cells (4 × 10^5^ cells per 50 μL) were injected into the inguinal mammary fat under the fourth right teat of the mouse.^[^ ^57^ ^]^ The tumors were monitored daily, and tumor measurements were taken every 2–3 days using digital calipers. The tumor volume *V* was estimated using Equation (1):

(1) $$V=\frac{l\;\times w^{2}}{2}$$

where *l* is the major axis of measurement and *w* is the minor axis of measurement. Mice observed with ulcerated tumors were immediately euthanized by inhalation of carbon dioxide according to university guidelines.

##### Administration of ReANCs and SWIR Imaging

Imaging was performed using an in‐house small animal SWIR imaging system described previously.^[^ ^22^, ^23^, ^24^, ^58^ ^]^ SWIR imaging was performed 11 and 17 days after tumor inoculation. One day before SWIR imaging, fur was removed from the mice using Nair hair removal cream if needed. If fur needed to be removed from around the tumor, electrical clippers were used instead of the hair removal cream to avoid irritation. On the day of imaging, mice were intravenously administered either CD8‐ReANCs or Iso‐ReANCs (100 μL; 50 mg HSA mL^−1^) via the tail vein. SWIR imaging was performed before ReANC injections to establish the background signal and 6 h after ReANC administration. During imaging, mice were anesthetized with 2%–3% isoflurane in oxygen delivered at a flow rate of 1 L min^−1^. The mice were scanned with a collimated continuous wave 980 nm laser with a 9.6 mm beam diameter and a power output of 1.7 W to excite the RENPs. A 512 × 640 pixel InGaAs camera (640HSX, Sensors Unlimited, Princeton, NJ) equipped with two 1150 nm long‐pass filters (Semrock, Rochester, NY), a 1497–1579 nm band‐pass filter (Semrock), and a 25 mm fixed focal length f/1.4 SWIR lens (StingRay Optics, Keene, NH) captured the SWIR light emitted from the animal. Real‐time imaging was performed with an exposure time of 33 ms per frame. Image data were acquired as.bin video files, and converted to a single.tiff image file based on the maximum pixel value from each frame using a custom Matlab script.^[^ ^24^ ^]^ To orient the resultant SWIR image to anatomical features on the mouse, a white light image was taken before SWIR imaging by illuminating the mouse with 1550 nm LED bulb.

##### SWIR Image Analysis

Quantitative analysis of SWIR images was performed using open‐source FIJI software (https://imagej.net/software/fiji/). Regions of interest (ROIs) were manually drawn around anatomical features on the white light image of a mouse. The mean pixel values were measured over the ROI from the corresponding SWIR image. To account for background noise and reflection of light from the mouse, the mean background SWIR signal of an ROI from the pre‐inject SWIR image was subtracted from the corresponding 6 h postinjection SWIR image. To account for inter‐experimental variation, data are presented as the difference between the CD8‐ReANC and Iso‐ReANC groups from independent experiments.

##### Immunohistochemistry

Tissue samples resected from BALB/c mice were fixed in 10% formalin for 24–48 h and then stored in 75% ethanol at 4 °C before tissue processing. The tissue samples were embedded in paraffin and cut into 5 μm sections using a microtome. The tissue slides were then deparaffinized with xylene, rehydrated, and placed in boiling citrate buffer at pH 6.0 for 20 min for antigen retrieval. The slides were washed with tris‐buffered saline (TBS), and blocked with a protein block solution (ab64226, Abcam) at 4 °C overnight. To stain for CTLs, recombinant rabbit anti‐mouse CD8α (clone EPR21769; Abcam) was diluted 1:250 in TBS + 2.5% BSA + 10% goat serum, added to the slides, and incubated overnight at 4 °C. Recombinant rabbit isotype control (clone EPR25A; Abcam) was used as a staining control. Ex vivo labeling with CD8‐ReANCs or Iso‐ReANCs was performed by diluting nanocomposites (0.5 mg HSA mL^−1^) in TBS + 2.5% BSA + 10% goat serum. For enzymatic immunohistochemistry, the slides were washed with TBS and treated with 0.3% hydrogen peroxide in TBS for 10 min at room temperature to block endogenous peroxidase, followed by additional washing. The slides were then treated with HRP‐conjugated secondary goat anti‐rabbit IgG H&L (Abcam ab97051) diluted 1:500 in TBS + 2.5% BSA + 10% goat serum for 1 h at room temperature. Slides were washed and treated with 3,3’‐diaminobenzidine substrate (Vector Laboratories, Newark, CA) according to the manufacturer's instructions. After washing with water, the slides were treated with Mayer's hematoxylin solution (Millipore Sigma) for 20 s and then washed with water for 5 min. The slides were then dehydrated with 99% isopropyl alcohol, followed by mounting with VectaMount permanent mounting medium (Vector Laboratories). Additionally, H&E staining of ex vivo liver and spleen samples was performed.

##### Statistical Analysis

All data in bar graphs are presented as the mean ± standard error of the means. Data in the form of box plots are shown from the 25th to 75th percentile, with a median line and a small square representing the mean. The box's whiskers represent the group's minimum and maximum value. All statistical analyses were performed using Origin 2021 software (Origin Lab, Northampton, MA), with α set to 0.05. Before statistical analysis, samples were screened for normality using a Shapiro‐Wilk test. For in vitro experiments, comparisons between two samples were made using a student's *t‐*test. Comparisons among multiple samples were first tested for homogeneity of variances using the Brown–Forsythe test. Then, a one‐way ANOVA test was performed, followed by a post hoc Tukey's test to correct for multiple comparisons. For in vivo experiments, statistical outliers were detected and omitted from subsequent analysis using a Grubb's test for outliers. Comparisons between groups with similar variances were made using a student's *t*‐test. If equal variances could not be assumed, comparisons between samples were made using a student's *t*‐test with Welch's correction. Comparisons of results across multiple in vivo experiments were made following the recommendations from Frommlet and Heinze on how to present and analyze data from various in vivo trials.^[^ ^59^ ^]^ The *p*‐values from individual experiments were combined using a weighted Z‐score^[^ ^60^ ^]^ as described in Equation (2):

(2) $$Z=\frac{\sum_{i=1}^{k}w_{i}Z_{i}\;}{\sqrt{\sum_{i=1}^{k}w_{i}^{2}}}$$

for *k* experiments, where *Z*_*i*_ is the Z‐score for the *i*th experiment and *w*_*i*_ is the weighting factor defined as the square root of the sample size. A *p*‐value below 0.05 was considered statistically significant.

## Conflict of Interest

The authors declare no conflict of interest.

## Supporting information

Supplementary Material

## Data Availability

The data that support the findings of this study are available from the corresponding author upon reasonable request.
