# Supplementary material for: Shortwave‐Infrared‐Emitting Nanoprobes for CD8 Targeting and In Vivo Imaging of Cytotoxic T Cells in Breast Cancer
Source: Adv Nanobiomed Res. 2023 Dec 5;4(2):2300092. doi: 10.1002/anbr.202300092 (PMC11566364; doi:10.1002/anbr.202300092)
Supplement: Supplementary file 1 — Supplementary Material [file ANBR-4-0-s001.pdf]

Supporting Information

Shortwave Infrared-Emitting Nanoprobes for CD8-Targeting and In Vivo Imaging of Cytotoxic T Cells in Breast Cancer

*Jay V. Shah, Jake N. Siebert, Xinyu Zhao, Shuqing He, Richard E. Riman, Mei Chee Tan, Mark C. Pierce, Edmund C. Lattime, Vidya Ganapathy, Prabhas V. Moghe**

Formulation	Hydrodynamic Diameter (nm)	Polydispersity Index
**ANC**	117.9 ± 1.5	0.133 ± 0.030
**IgG-ANC**	118.9 ± 2.2	0.100 ± 0.011
**ReANC**	133.3 ± 2.4	0.141 ± 0.008
**CD8-ReANC**	138.5 ± 4.0	0.143 ± 0.027
**Iso-ReANC**	145.1 ± 4.6	0.168 ± 0.032
**IgG-ReANC**	141.8 ± 3.1	0.155 ± 0.020

Table S1. Characterization of nanocomposite formulations. The IgG prefix represents the means of pooled data from CD8-functionalized and Iso-functionalized nanocomposites. No significant differences in hydrodynamic diameter were observed among the different ReANC formulations.

Parameter	Estimated Value	Comments on Calculation
**Protein Mass of Nanocomposite**	1.16 x 10^{-12} mg	ANCs are assumed to be spherical (diameter = 118 nm) with a protein density of 1.35 g/cm^3
**Nanocomposite Concentration**	5.82 x 10^{13} ANCs from 70 mg HSA	Calculated based on the mass of final HSA (yield = 96.6% as determined by BCA) divided by the protein mass of nanocomposites
**Antibody Concentraiton**	3.23 μg IgG per mg HSA	Calculated based on the conjugation efficiency (11.3% [12.7% for anti-CD8 and 9.9% for isotype control]) as calculated by ELISA with a loading density of 28.6 ug IgG/mg HSA
**Antibody to Nanocomposite Ratio**	9.07 x 10^{14} IgG per 5.82 x 10^{13} ANCs	Calculated based on the nanocomposite yield synthesized from 70 mg HSA and a loading density of 28.6 μg HSA / mg HSA
**Antibodies per Nanocomposite**	~15-16 antibodies per ANC or ReANC	Estimated with the ratio of antibodies to nanocomposites

Table S2. Estimation of the number of antibodies per ANC and ReANC based on experimental values.

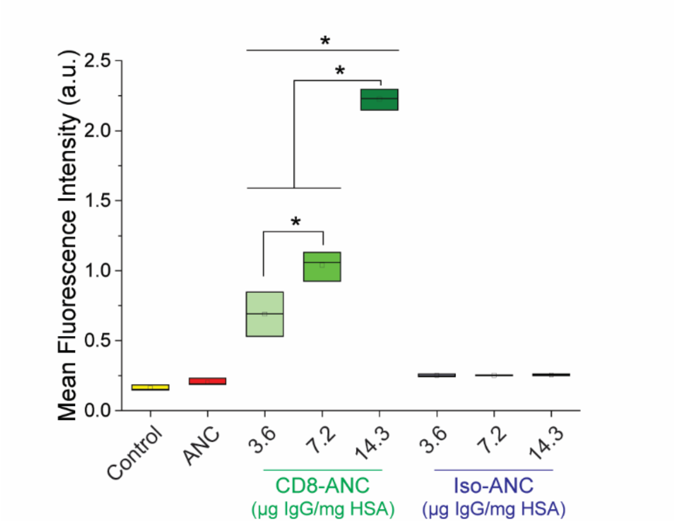

Figure S1. Differential antibody loading densities for CTL targeting. Primary mouse CTLs were treated with PBS as a negative control or FITC-labeled ANCs, CD8-ANCs, or Iso-ANCs for 1 h

at room temperature. ANC binding was assessed by flow cytometry. Functionalizing ANCs with anti-CD8α increased ANC uptake within the range of 3.6-14.2 μg IgG per mg HSA, as assessed by mean fluorescence intensity (MFI). Among the CD8-ANC groups, those loaded with 14.2 μg IgG per mg HSA had a higher MFI than ANCs loaded with 3.6 μg IgG per mg HSA and 7.2 μg IgG per mg HSA ($p<0.0001$). T cells with CD8-ANCs loaded with 7.2 μg IgG per mg HSA had a higher MFI than those with CD8-ANCs loaded with 3.6 μg IgG per mg HSA ($p=5.216 \times 10^{-4}$). Compared to the cell-only control, no increase in MFI was seen with ANCs ($p=0.9955$) or Iso-ANCs ($p=0.7844$, $p=0.7747$, $p=0.7549$ for loading densities of 3.6, 7.2, and 14.3 μg IgG per mg HSA, respectively). The box plots represent the means from three independent experiments with three technical replicates per experiment. Analysis was performed using a one-way ANOVA and a *post hoc* Tukey's test for multiple comparisons. Asterisks denote statistical significance.

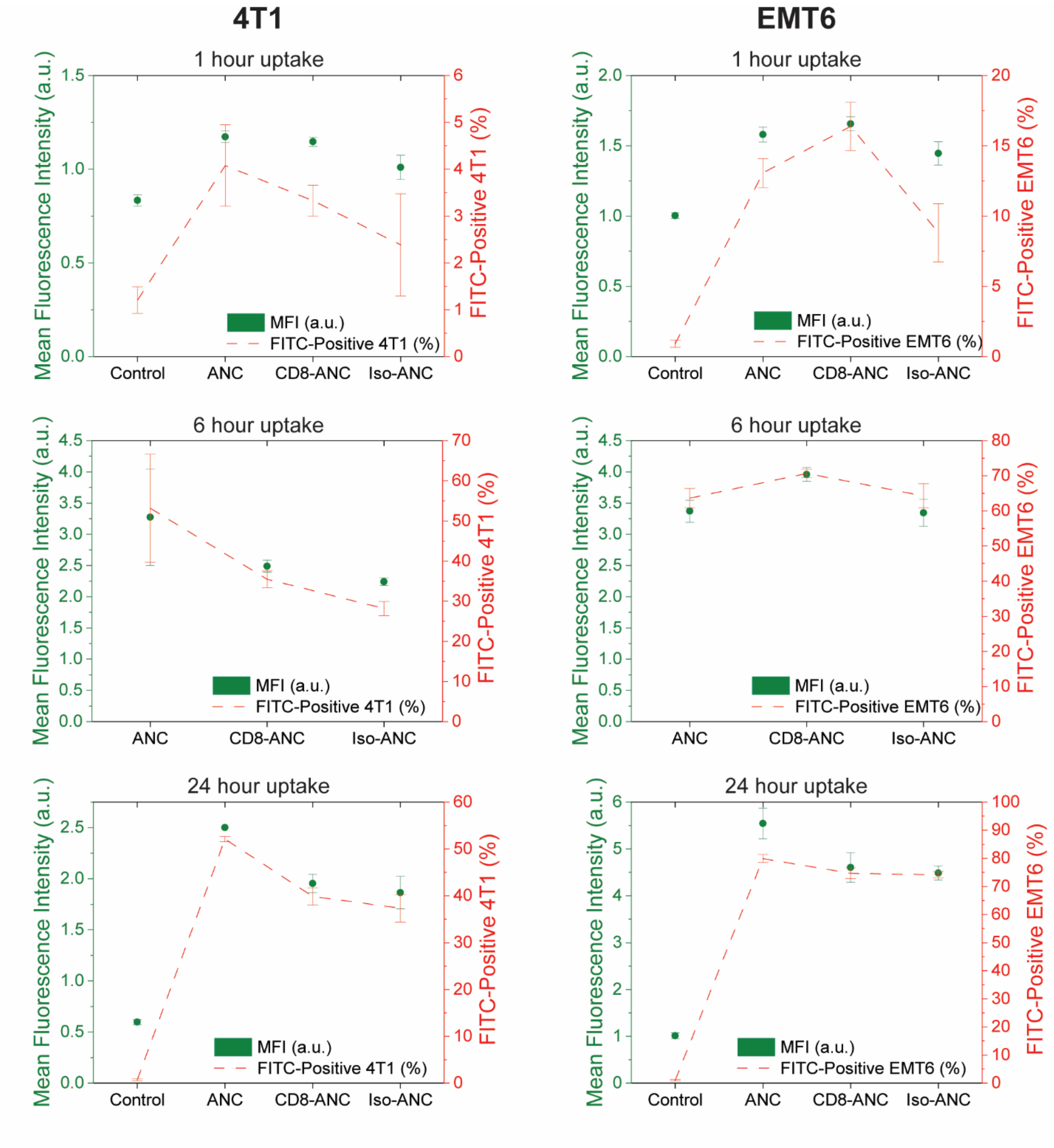

Figure S2. CD8-targeted ANCs are not taken up by tumor cells, compared to control ANCs. $5x10^4$ 4T1 (left) or EMT6 (right) cells in 96 well-plates were treated with FITC-labeled ANCs, CD8-ANCs, or Iso-ANCs (0.5 mg HSA mL^{-1}) for 1, 6, and 24 h, and then analyzed for ANC uptake by flow cytometry (*n*=1 with three technical replicates). No differences in uptake between CD8-ANCs and Iso-ANCs was observed for either cell line regarding MFI or the number of cells positive for the FITC signal.

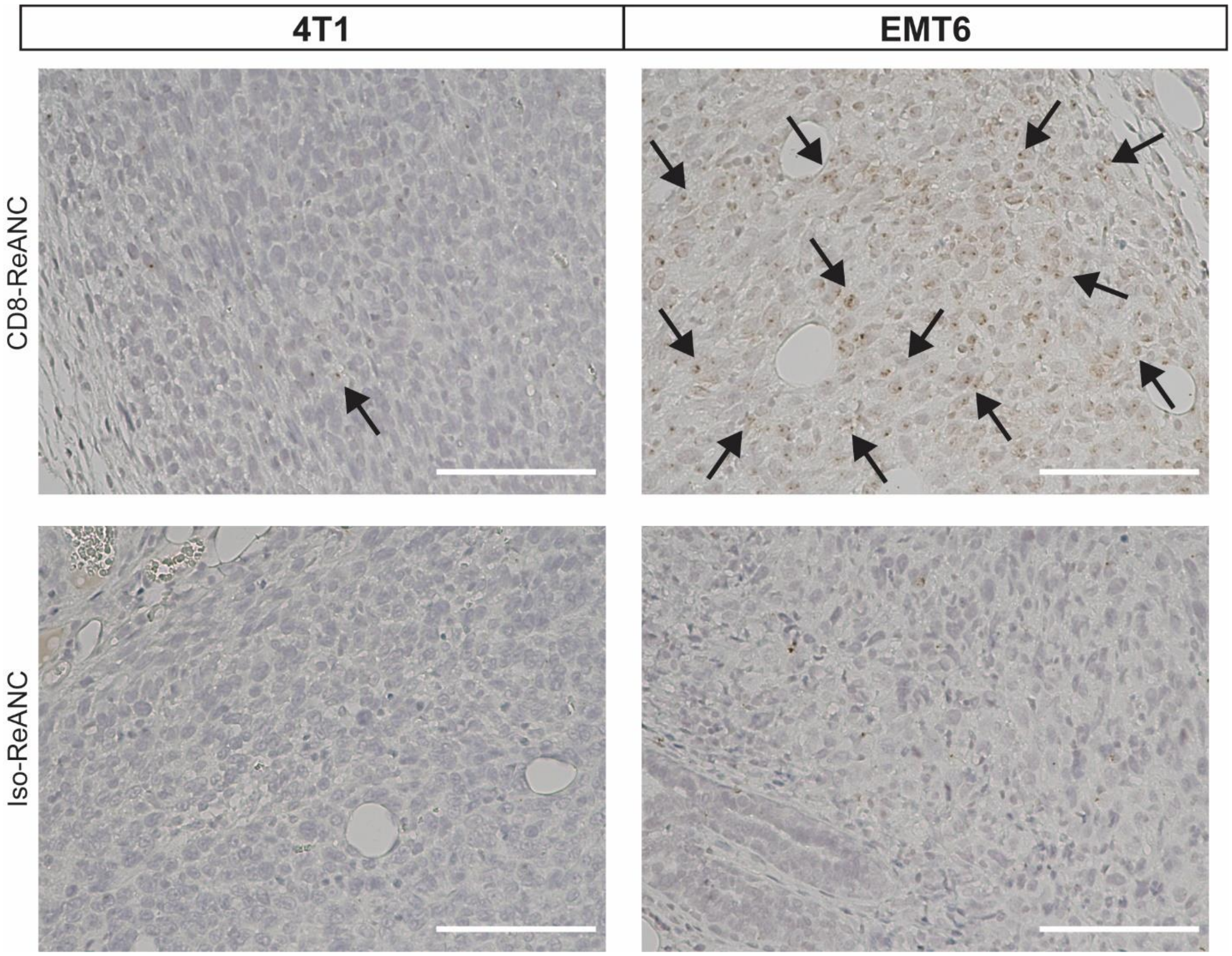

Figure S3. CD8-ReANCs stain for T cells on tumor slides, but Iso-ReANCs do not. Tumor sections were stained with CD8-ReANCs to examine the specificity of targeted probes. Iso-ReANCs were used as a control for background staining. Little staining is present with CD8-ReANCs in a non-immunogenic 4T1 tumor, while there was positive staining in an EMT6 tumor, known be infiltrated with $CD8^+$ T cells (see **Figure S6**).

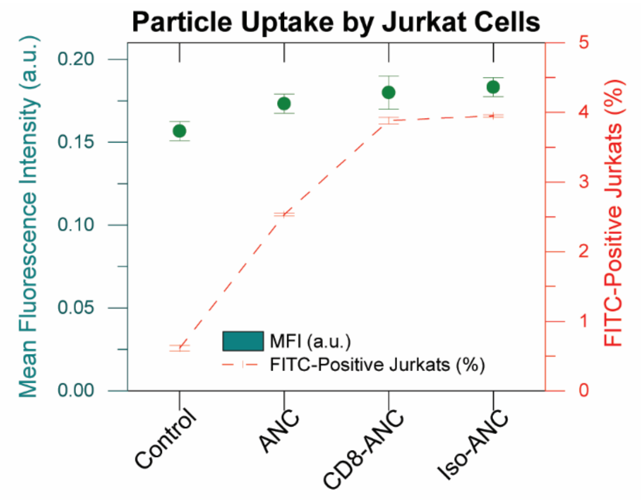

Figure S4. Mouse CD8-targeted ANCs are not taken up by human T cells that do not express CD8. 1x10^5 Jurkat cells, a human CD8$^-$ T cell line, were treated with FITC-labeled ANCs, CD8-ANCs, or Iso-ANCs (0.5 mg HSA mL^{-1}) and analyzed for ANC uptake by flow cytometry (n=1 with three technical replicates). There were no differences in uptake between CD8-ANCs and Iso-ANCs regarding MFI or the number of cells positive for the FITC signal.

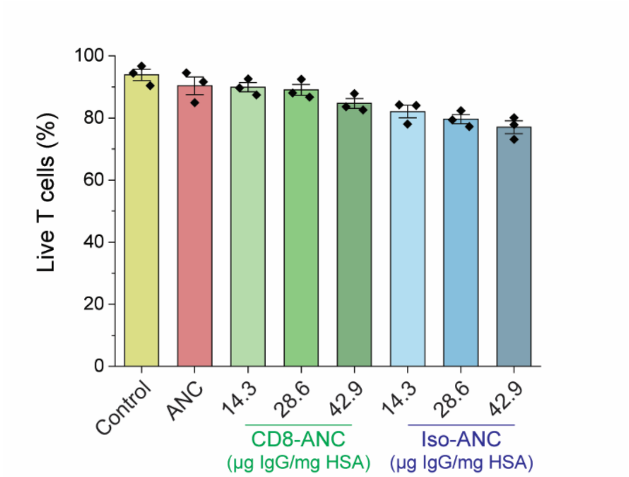

Figure S5. Treatment of primary mouse CTLs with CD8-ANCs does not affect T cell viability. 1x10^5 T cells in 100 µL of RPMI media supplemented with 10% FBS and 1% P/S were treated with PBS as a cell-only control or ANCs with varying ligand density at a final concentration of 0.5 mg HSA mL^{-1} for 1 h and then treated with DAPI for analysis by flow cytometry (**Figure 3**). The percentage of live cells was quantified. No reduction in cell viability was seen for T cells

treated with CD8-ANCs of any ligand density compared to the control. Data in the bar graph represents the mean ± SEM from three independent experiments with three technical replicates per experiment.

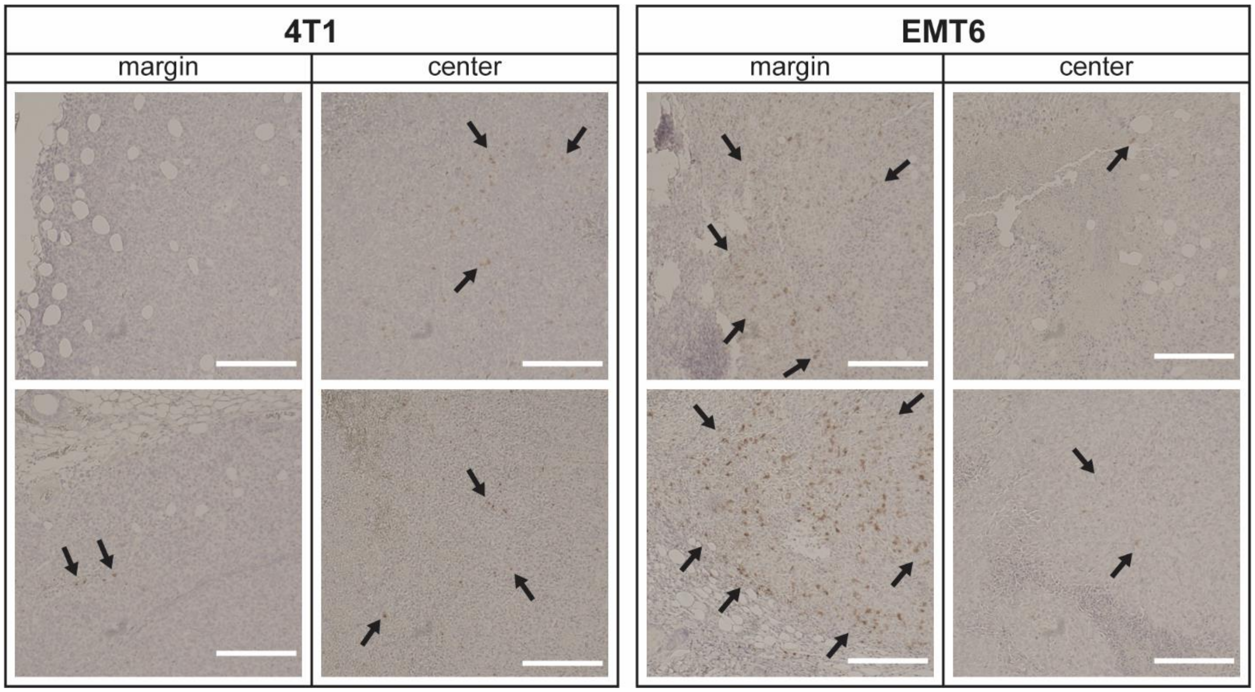

Figure S6. 4T1 and EMT6 tumors differ in CTL infiltration. Formalin-fixed, paraffin-embedded tumor sections were stained for CD8 (brown) to visualize the spatial distribution of CTLs and counterstained with hematoxylin (blue). In 4T1 tumors (left), few $CD8^+$ T cells were present in the invasive margin. More CTLs were present in the tumor center, but their distribution was sporadic and spread out. In EMT6 tumors (right), several clusters of CTLs were observed at the invasive margins of the tumors. However, very few CTLs were observed in the tumor center. Black arrows point to some examples of CD8 staining. Scale bar = 200 μm.

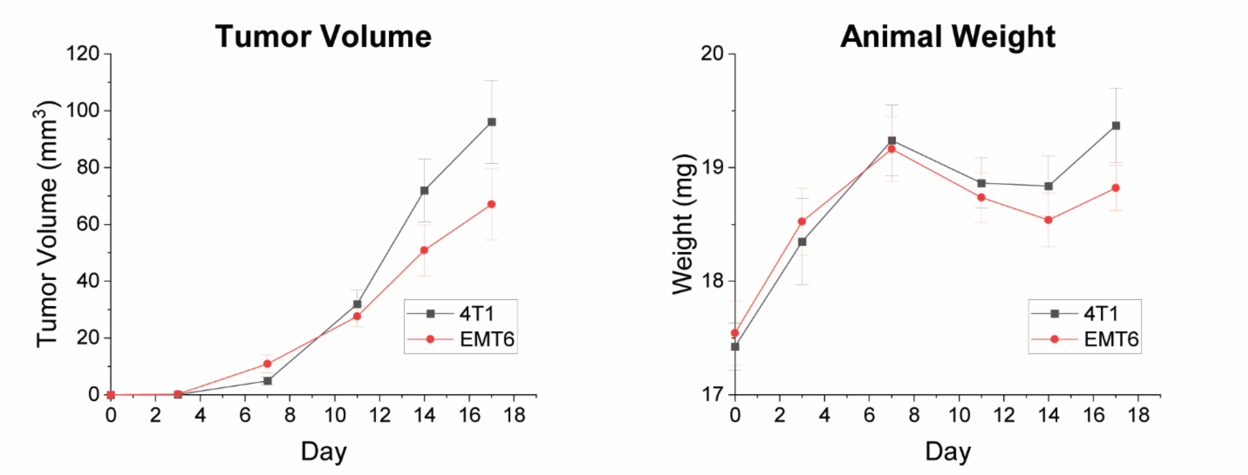

Figure S7. The 4T1 and EMT6 tumor models do not differ in growth kinetics. $4x10^5$ 4T1 or EMT6 cells were injected into the 4th right teats of Balb/c mice to establish the orthotopic mammary fat pad breast cancer models. No statistical differences were seen between the models regarding tumor volume (left). SWIR imaging was performed 11 and 17 days after tumor inoculation. On day 11, 4T1 tumors were 32.0 ± 4.8 mm^3 in volume, and EMT6 tumors had an average volume of 27.6 ± 3.7 mm^3 (p=0.4723). On day 17, 4T1 tumors had an average volume of 96.0 ± 14.6 mm^3, while EMT6 tumors were 67.1 ± 12.6 mm^3 in volume (p=0.1376). No impact was observed on animal weight in response to growing tumors for 4T1 or EMT6 mice (left).

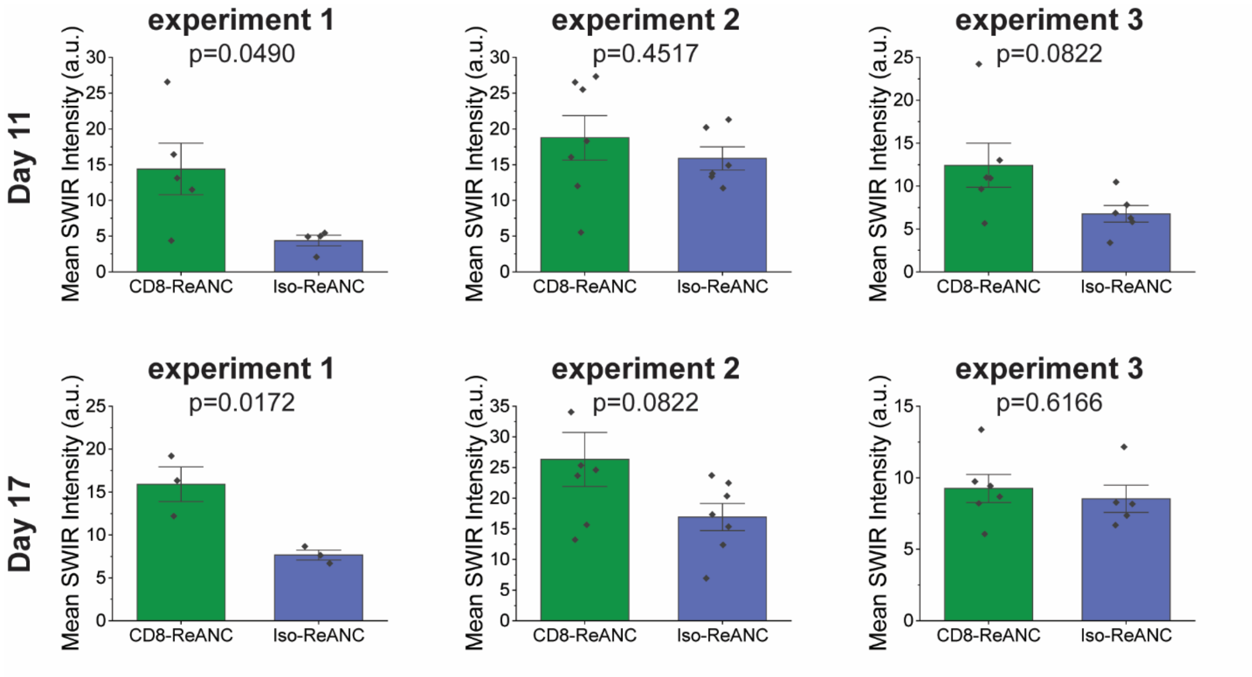

Figure S8. SWIR imaging data from three independent experiments in EMT6-bearing mice show that the SWIR signal is generally greater in the tumors of mice that received CD8-ReANCs compared to Iso-ReANCs. The bar graphs represent the data from the independent SWIR imaging experiments used to generate **Figure 5**. Each data point is the mean SWIR intensity over a region of interest drawn over the mouse's tumor. For the first imaging timepoint performed 11 days after tumor inoculation, the two-tailed *p* values were 0.0490, 0.4517, and 0.0822 for experiment 1 (*n*=5 CD8-ReANC, *n*=4 Iso-ReANC), experiment 2 (*n*=7 CD8-ReANC, *n*=6 Iso-ReANC), and experiment 3 (*n*=6 CD8-ReANC, *n*=6 Iso-ReANC), respectively. The *p* values for day 17 were 0.0172, 0.0822, and 0.6166 for experiment 1 (*n*=3 CD8-ReANC, *n*=3 Iso-ReANC), experiment 2 (*n*=7 CD8-ReANC, *n*=7 Iso-ReANC), and experiment 3 (*n*=6 CD8-ReANC, *n*=5 Iso-ReANC), respectively. Comparisons were made using a student's *t*-test, unless the sample variances were significantly different, as was the case for experiment 1 day 11 and experiment 3 day 11. For these experiments, comparisons were made using a student's *t*-test with Welch's correction for unequal variances. * $p<0.05$.

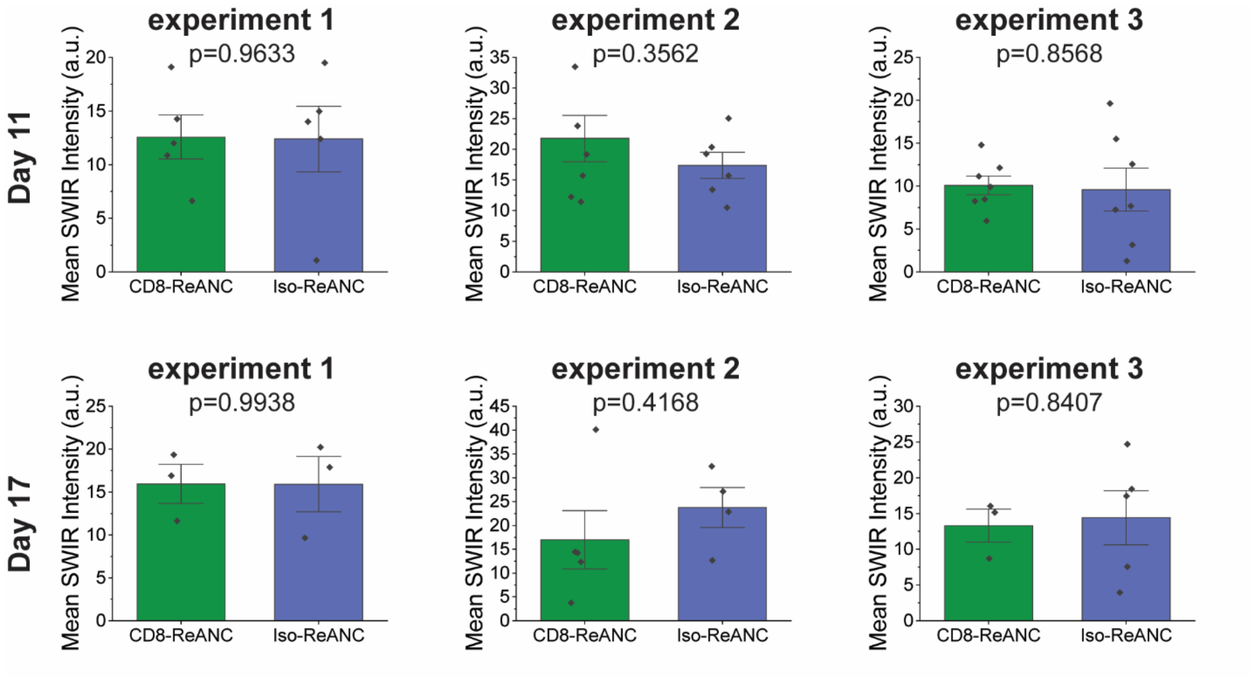

Figure S9. SWIR imaging data from three independent experiments in 4T1-bearing mice show that the SWIR signal is generally similar in tumors of mice that received CD8-ReANCs and Iso-ReANCs. The bar graphs represent the data from the independent SWIR imaging experiments used generated in **Figure 6**. Each data point is the mean SWIR intensity over a region of interest drawn over the mouse's tumor. For the first imaging timepoint performed 11 days after tumor inoculation, the two-tailed p values were 0.9633, 0.3562, and 0.8568 for experiment 1 (n=5 CD8-ReANC, n=5 Iso-ReANC), experiment 2 (n=7 CD8-ReANC, n=6 Iso-ReANC), and experiment 3 (n=7 CD8-ReANC, n=7 Iso-ReANC), respectively. The p values for day 17 were 0.9938, 0.4168, and 0.8407 for experiment 1 (n=3 CD8-ReANC, n=3 Iso-ReANC), experiment 2 (n=5 CD8-ReANC, n=4 Iso-ReANC), and experiment 3 (n=3 CD8-ReANC, n=5 Iso-ReANC), respectively. Comparisons were made using a student's t-test. * $p<0.05$.

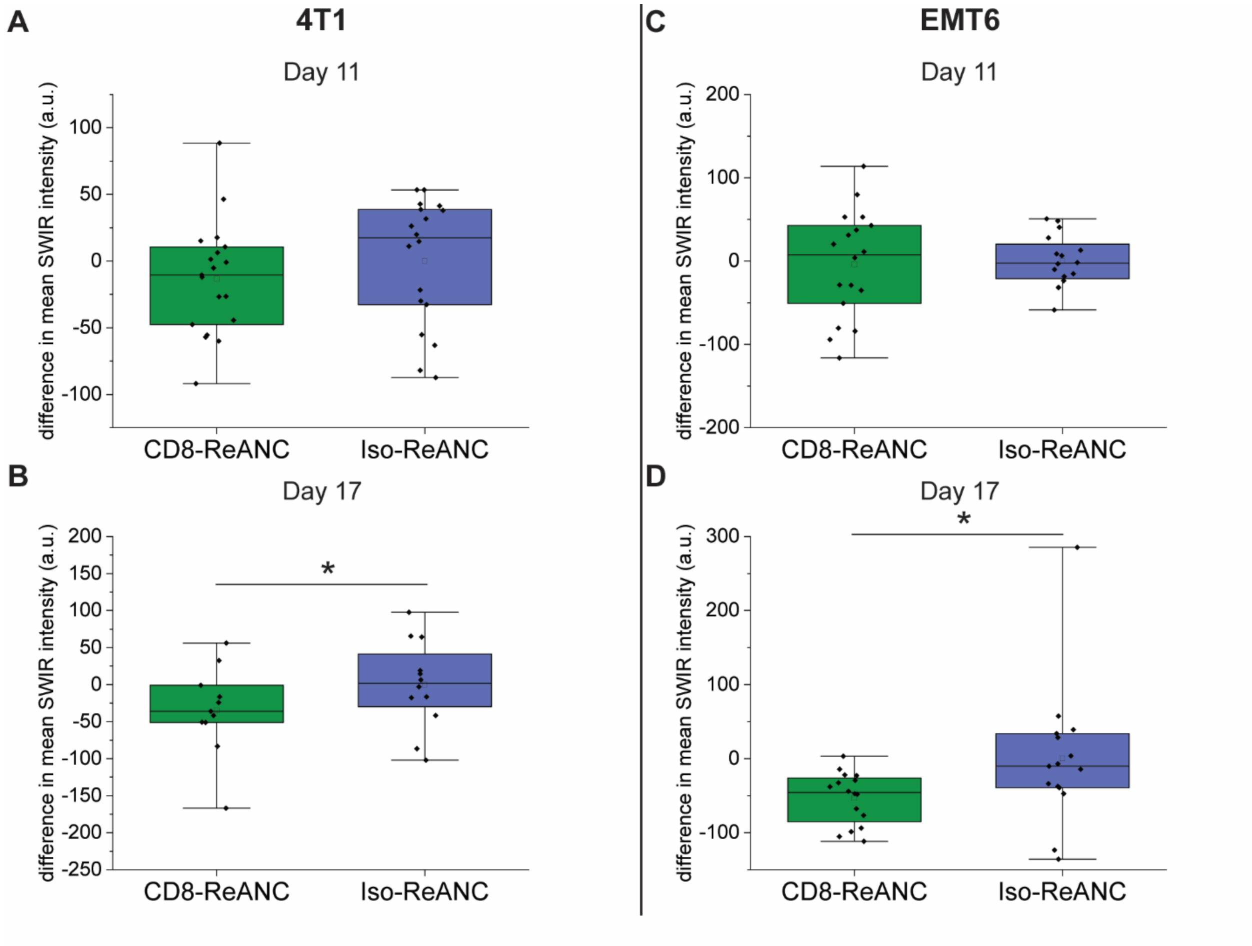

Figure S10. SWIR signal in the livers of mammary fat pad tumor-bearing mice after administration of CD8-ReANCs or Iso-ReANCs. The mean SWIR intensity was quantified over an ROI drawn around the general area where the mouse's liver was expected to be. Three independent *in vivo* imaging experiments were performed. Data points in the box plots represent the difference between the mean pixel intensity from each liver and the mean SWIR intensity from the Iso-ReANC group for each experiment. A) There was no difference in liver SWIR signal in 4T1 mice on day 11 across the three experiments (p=0.3029). The two-tailed p values were 0.1620, 0.9152, and 0.96963 for experiment 1 (n=5 CD8-ReANC, n=5 Iso-ReANC), experiment 2 (n=7 CD8-ReANC, n=6 Iso-ReANC), and experiment 3 (n=7 CD8-ReANC, n=7 Iso-ReANC), respectively. B) On day 17, there was significantly more signal from the livers of 4T1-bearing mice that received Iso-ReANC compared to CD8-ReANCs over the three experiments (p=0.0231), indicating there may be an increased clearance of the Iso-ReANCs. The p values were 0.0158, 0.1994, and 0.6896 for experiment 1 (n=3 CD8-ReANC, n=3 Iso-

ReANC), experiment 2 (*n*=5 CD8-ReANC, *n*=4 Iso-ReANC), and experiment 3 (*n*=3 CD8-ReANC, *n*=5 Iso-ReANC), respectively. C) Similar SWIR signal was observed in the livers of EMT6-bearing mice that received CD8-ReANCs compared to Iso-ReANCs over three experiments (p=0.2905). The p values were 0.8940, 0.4621, and 0.3689 for experiment 1 (*n*=5 CD8-ReANC, *n*=4 Iso-ReANC), experiment 2 (*n*=7 CD8-ReANC, *n*=6 Iso-ReANC), and experiment 3 (*n*=6 CD8-ReANC, *n*=6 Iso-ReANC), respectively. D) On day 17, as with the 4T1 livers, there was significantly more signal from the livers of EMT6-bearing mice that received Iso-ReANCs compared to CD8-ReANCs over the three experiments (p=0.0260). The p values for day 17 EMT6 spleens were 0.5017, 0.2977, and 0.0388 for experiment 1 (*n*=3 CD8-ReANC, *n*=3 Iso-ReANC), experiment 2 (*n*=7 CD8-ReANC, *n*=7 Iso-ReANC), and experiment 3 (*n*=6 CD8-ReANC, *n*=5 Iso-ReANC), respectively. * p<0.05.

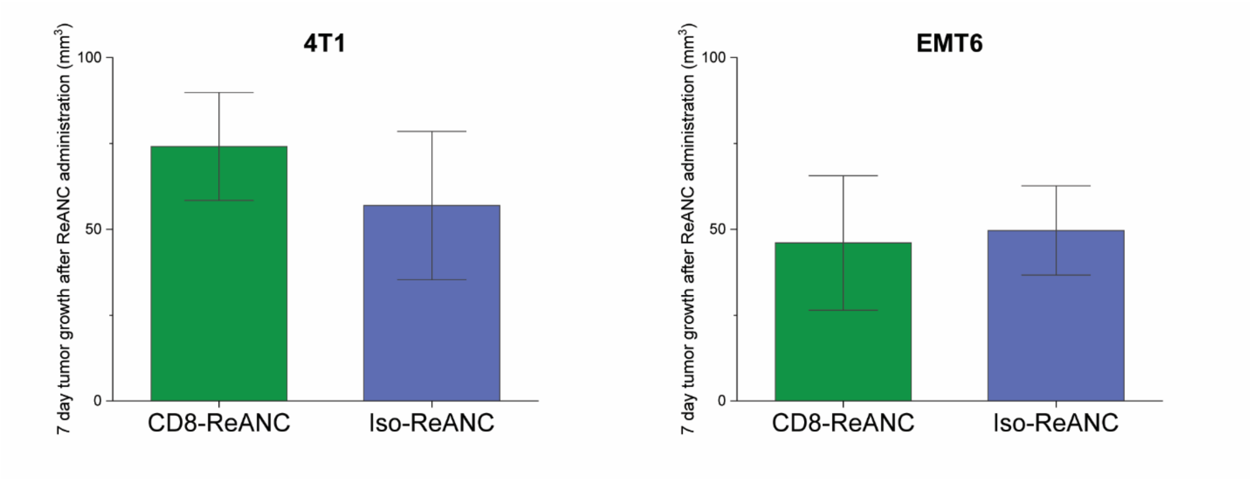

Figure S11. ReANC formulations do not influence tumor growth. One week after administration of ReANCs, no significant differences were observed in tumor growth between mice administered CD8-ReANCs or Iso-ReANCs in the 4T1 model (p=0.5218) or EMT6 model (p=0.8746). Comparisons were made using a student's *t*-test.

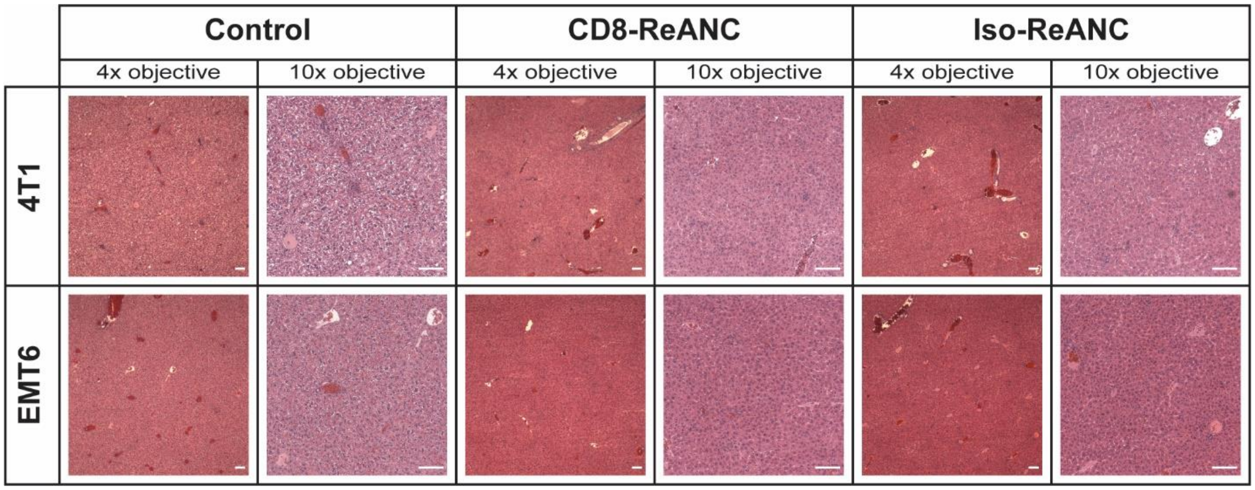

Figure S12. Hematoxylin and eosin (H&E) images of livers show no apparent changes in tissue architecture after administration of ReANCs compared to untreated controls. Representative H&E images of livers at two magnification levels are provided for mice bearing 4T1 or EMT6 tumors. Similar hepatic organization and vasculature are observable in the livers of mice that received CD8-ReANCs and Iso-ReANCs compared to the ReANC-free controls. Scale bar = 100 μm.

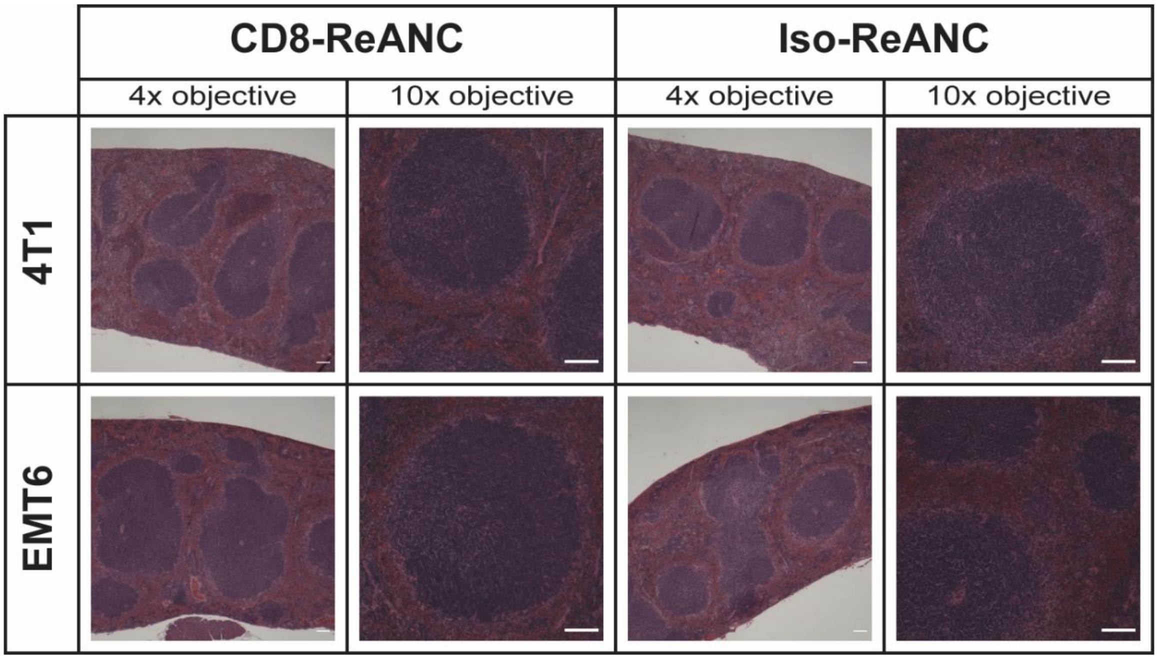

Figure S13. Hematoxylin and eosin (H&E) images show mostly typical splenic architecture after treatment with ReANCs. Representative H&E images of spleens at two magnification levels are provided for mice bearing 4T1 or EMT6 tumors. Scale bar = 100 μm.
